# Aging-associated immune and stromal programs mark interferon- and remodeling-linked spatial contexts in psoriatic skin

**DOI:** 10.3389/fimmu.2026.1903929

**Published:** 2026-08-11

**Authors:** Xuejingzi Wu, Mengwen He, Hongxiang Chen, Lu Gan

**Affiliations:** 1 Department of Dermatology, Zhongnan Hospital of Wuhan University, Wuhan, China; 2 Department of Dermatology, the Fifth Affiliated Hospital of Sun Yat-sen University, Zhuhai, China

**Keywords:** immune-aging, psoriasis, skin aging, spatial transcriptomics, stromal remodeling

## Abstract

**Introduction:**

Psoriasis is a chronic immune-mediated inflammatory skin disease characterized by spatially organized epithelial, stromal, vascular, and immune interactions. Although aging is associated with inflammatory activation and tissue remodeling in skin, it remains unclear how aging-associated transcriptional programs are positioned within psoriatic tissue architecture and whether stromal cells with senescence-associated features retain inflammatory responsiveness.

**Methods:**

We integrated a normal aging human skin single-cell RNA-seq dataset with discovery and external psoriasis spatial transcriptomic datasets. Three compartment-informed aging signatures—global old-up, stromal-aging, and immune-aging—were defined and projected onto psoriatic tissue. Sensitivity analyses included an overlap-removed immune-aging score and immune/APC-adjusted analyses. Human foreskin fibroblasts were used as a reductionist system to test whether doxorubicin-induced senescence-like stress was compatible with IFNγ-responsive antigen-presentation-associated and chemokine programs.

**Results:**

Aging-associated signals were not uniformly distributed across psoriatic lesions. Global old-up and immune-aging programs were higher in APC/immune-enriched and immune-inflammatory domains, whereas stromal-aging localized to fibro-inflammatory and extracellular matrix-remodeling domains. Score-defined immune-aging-high regions showed IFN-linked chemokine, inflammatory, and immune-activation features, while stromal-aging-high regions were dominated by collagen organization and extracellular matrix remodeling. At the tissue-section level, immune-aging activity was increased in lesional psoriasis and was associated with IFN gamma response and antigen-presentation activity, with similar patterns observed using the overlap-removed immune-aging score. External spatial transcriptomic analysis reproduced selected antigen-presentation- and interferon-linked features. In HFF-1 fibroblasts, doxorubicin induced a senescence-like DNA damage-associated state, whereas IFNγ induced HLA-ABC, B2M, CD74, CXCL9, and surface HLA-DR. These IFNγ-responsive programs remained detectable after doxorubicin-induced stress.

**Discussion:**

Aging-associated transcriptional programs are spatially organized within psoriatic skin through distinct immune- and stromal-associated tissue contexts. The findings support the coexistence of senescence-like stromal stress and IFNγ-responsive immune activation but do not establish a causal relationship between these processes.

## 1 Introduction

Psoriasis is a chronic immune-mediated inflammatory skin disease characterized by epidermal hyperplasia, altered keratinocyte differentiation, immune-cell infiltration, and sustained cytokine signaling within the skin (1). Current studies have defined several central pathogenic pathways, particularly the IL-23/IL-17 axis and keratinocyte–immune-cell inflammatory feedback loops (2). More recent single-cell and spatial transcriptomic analyses have further shown that psoriatic lesions are not simply collections of activated immune cells or hyperproliferative keratinocytes, but spatially organized tissues in which epithelial, stromal, vascular, and immune compartments interact locally (3). These advances have substantially improved our understanding of the cellular and spatial organization of psoriatic inflammation (4). However, most studies still focus on disease-associated inflammatory programs themselves, while less is known about how broader tissue-state modifiers, such as aging-related transcriptional programs, are positioned within psoriatic tissue architecture. This is an important gap because aging may influence inflammatory skin disease through multiple compartments, including epidermal stress responses, stromal remodeling, vascular changes, and immune activation. Defining where these aging-associated programs localize within psoriatic lesions may therefore help clarify whether aging-related biology is broadly distributed across diseased skin or concentrated within specific immune or stromal tissue contexts.

This question requires aging to be considered not only as a patient-level demographic feature, but also as a set of tissue and cellular programs that may intersect with psoriatic inflammation. Skin aging is accompanied by changes in epidermal differentiation, barrier function, dermal extracellular matrix organization, vascular structure, cellular senescence, and chronic low-grade inflammatory activation (5). These processes are often discussed as inflammaging, immunosenescence, or senescence-associated tissue remodeling, but they are unlikely to act uniformly across the skin. Aging-related changes in keratinocytes, fibroblasts, endothelial cells, and immune cells may each have different relationships to psoriasis-associated inflammation. For example, an epidermal aging program may overlap with barrier stress or altered differentiation, a stromal-aging program may reshape extracellular matrix and tissue architecture, and an immune-aging program may affect cytokine response, antigen presentation, or inflammatory recruitment (6). Therefore, the relevant question is not simply whether aged skin differs from young skin, but whether distinct aging-related programs map to specific tissue domains within psoriatic lesions. Answering this requires an approach that can first separate aging signals by cellular compartment and then examine how those signals are spatially organized in diseased skin.

Single-cell and spatial transcriptomic approaches are well suited to address this problem because they provide complementary information. Single-cell RNA sequencing can define aging-associated programs within specific cellular compartments, helping to distinguish broad old-skin transcriptional changes from stromal- or immune-associated aging signals (7). Spatial transcriptomics can then determine whether these programs are diffusely distributed across diseased tissue or concentrated within particular tissue domains. This distinction is especially important in psoriasis, where inflammatory signaling is organized through local interactions among keratinocytes, fibroblasts, immune cells, and vascular-associated cells (8). However, most spatial analyses of psoriasis have focused on disease-associated cell states and inflammatory pathways themselves (4). It remains less clear whether aging-associated programs can be mapped onto psoriatic tissue architecture and whether they identify specific immune or stromal spatial contexts within lesions. Integrating aging skin single-cell data with psoriasis spatial transcriptomics therefore provides a way to move from cell-type-specific aging programs to tissue-domain-level organization in inflammatory skin disease.

In this study, we asked whether aging-associated transcriptional programs are spatially organized within psoriatic skin and whether they correspond to distinct inflammatory or stromal tissue contexts. We first defined global, stromal, and immune-associated aging programs from an aging human skin single-cell RNA-seq dataset and then projected these programs onto psoriasis spatial transcriptomic cohorts. This approach allowed us to examine aging-related biology not as a single patient-level variable, but as compartment-informed transcriptional programs positioned within diseased tissue architecture. We found that aging-associated programs were not uniformly distributed across psoriatic tissue. Instead, immune-aging-associated activity was linked to APC/immune and immune-inflammatory regions and was associated with interferon response, antigen-presentation activity, chemokine signaling, and immune-cell-enriched spatial contexts, whereas stromal-aging signals localized to fibro-inflammatory and ECM-remodeling regions. We further used an external spatial cohort and a simplified fibroblast experiment to evaluate selected features of this correlative spatial model. Together, these analyses identify spatially distinct immune- and stromal-associated aging programs in psoriatic skin and provide a framework for studying how aging-related tissue states may intersect with chronic cutaneous inflammation.

## 2 Materials and methods

### 2.1 Data collection

Three public transcriptomic datasets were used in this study. The aging skin single-cell RNA-seq dataset GSE130973 was used to define aging-associated transcriptional programs in normal human skin (7). This dataset included two young donors and three old donors. The young samples were y1 and y2, corresponding to 25- and 27-year-old donors, respectively. The old samples were o1, o2, and o3, corresponding to 53-, 70-, and 69-year-old donors, respectively. After preprocessing and filtering, 15,780 cells were retained for analysis, including 5,681 young cells and 10,099 old cells.

The discovery psoriasis spatial transcriptomic analysis was performed using GSE206391 (9). Although the original dataset contains multiple patients, the processed H5 spatial transcriptomic file used in this study contained samples from Patient 1, Patient 10, and Patient 13. All 12 available sections from these three patients were included, comprising lesional and non-lesional sections and 9,830 retained spots after preprocessing. Histology images were used as morphological references, while quantitative analyses were performed on spatial transcriptomic spots.

The external support analysis was performed using GSE202011, an independent spatial transcriptomic dataset containing healthy skin, cutaneous psoriasis, and psoriatic arthritis skin samples (4). This dataset included 24,227 spatial spots from 30 tissue sections across 9 individuals. The external cohort contained healthy skin, psoriasis non-lesional, psoriasis lesional, psoriatic arthritis non-lesional, and psoriatic arthritis lesional sections.

### 2.2 Aging skin single-cell RNA-seq analysis and aging signature definition

The aging skin single-cell RNA-seq dataset GSE130973 was analyzed in R using Seurat (10). Cells were visualized by UMAP and annotated into broad skin compartments using canonical marker expression. The major annotated compartments included keratinocytes, fibroblast/stromal cells, immune cells, endothelial cells, melanocytes, and pericyte/smooth muscle cells. When pre-existing broad annotation was not available, broad cell-type labels were assigned by calculating canonical marker module scores for each cluster and assigning the cluster to the marker program with the highest average score. Clusters with low-confidence marker separation were assigned as low-confidence or other categories.

Cell-type composition was calculated for each sample as the fraction of cells assigned to each broad cell type. Pooled old-versus-young differential expression analysis was performed to obtain a global overview of aging-associated transcriptional shifts. Genes were considered aging-associated using an adjusted P-value threshold of 0.05 and an absolute average log2 fold-change threshold of 0.5. Because pooled differential expression can be affected by cell-type composition, these results were interpreted as a global aging overview rather than as cell-type-specific differential expression.

Three aging-associated signatures were defined from the aging skin single-cell analysis: global old-up, stromal-aging, and immune-aging. Pooled old-versus-young differential expression first identified a broad old-biased gene pool containing genes with adjusted P < 0.05 and positive old-versus-young fold change. A stricter global old-up signature was then defined using adjusted P < 0.05 and average log2 fold change > 0.5 and was used for downstream spatial projection. The stromal-aging signature was derived from old-associated genes enriched in stromal compartments and extracellular matrix-related aging features. The immune-aging signature was derived from old-associated genes enriched in immune compartments. The final downstream signatures contained 683 genes for the strict global old-up signature, 201 genes for stromal-aging, and 115 genes for immune-aging. For sensitivity analysis, canonical immune-overlap genes were removed from the immune-aging signature to generate an overlap-removed immune-aging score. Expression-matched random gene-set controls were also generated to test whether the observed aging-associated score patterns could be reproduced by random gene sets of comparable size and expression level. Module scores were calculated using Seurat AddModuleScore through a custom wrapper function that retained only genes present in the analyzed object and stored the resulting module score as a metadata column. For the aging skin single-cell analysis, old-minus-young score differences were summarized within each broad cell type to determine which compartments contributed most strongly to each aging-associated program.

### 2.3 Discovery psoriasis spatial transcriptomic analysis

The discovery spatial transcriptomic analysis was performed using the processed GSE206391 spatial expression matrix. Spatial expression data were imported into R and analyzed as a Seurat object. Metadata columns were used to identify sample, patient, lesion status, spatial domain annotation, and spatial coordinates. Patient labels were corrected to Patient 1, Patient 10, and Patient 13 based on sample identifiers. The 12 sections included P15509_1001, P15509_1002, P15509_1003, P15509_1004, P16357_1033, P16357_1034, P16357_1035, P16357_1036, P16357_1045, P16357_1046, P16357_1047, and P16357_1048. Spatial coordinates were rescaled within each sample for visualization.

Spatial spots were annotated into tissue-associated transcriptional domains using canonical epithelial, stromal, immune, vascular, and inflammatory marker programs. The initial domain annotation included psoriatic keratinocyte-enriched, differentiated/stress keratinocyte, immune-enriched inflammatory, ECM-stromal fibroblast, fibro-inflammatory interface, pericyte/VSMC-rich vascular, APC/immune-enriched, appendage-associated epithelial, melanocytic/appendage-associated, adipose-associated stromal, and other/unannotated domains. These labels were interpreted as spot-level tissue states rather than purified cell identities.

The global old-up, stromal-aging, and immune-aging signatures defined from GSE130973 were projected onto GSE206391 spatial spots using module-score analysis. Domain-level mean scores were calculated by averaging spot-level module scores within each marker-defined spatial domain, and section-level summaries were generated by averaging scores within each domain for each tissue section. To define score-defined aging-high spatial regions, immune-aging and stromal-aging scores were scaled within each tissue section. Spots with high immune-aging activity were assigned to an immune-aging-high region class, spots with high stromal-aging activity were assigned to a stromal-aging-high region class, and the remaining spots were retained as other regions. Marker-defined spatial domains were then overlaid *post hoc* to summarize where the score-defined high regions were located. Threshold-sensitivity analyses using alternative top-score cutoffs were performed to evaluate whether high-region assignments were robust to cutoff choice.

Unbiased marker discovery was performed using Seurat FindMarkers. Score-defined immune-aging-high regions were compared with stromal-aging-high and other regions, and stromal-aging-high regions were similarly compared with immune-aging-high and other regions. Marker testing was performed with no log-fold-change threshold and a minimum detection fraction of 0.05. Marker genes were ranked by adjusted P value and log2 fold-change. Pathway enrichment analysis was performed on region-enriched marker genes using Hallmark and Gene Ontology Biological Process gene sets from MSigDB. Enrichment was summarized using odds ratio, false discovery rate, and overlap size.

Targeted inflammatory and immune-related gene modules were also scored in spatial spots. Modules included IFN gamma response, cytokine/chemokine signaling, antigen presentation, CXCL9/CXCL10 chemokine axis, T-cell cytotoxicity, macrophage/DC activation, and TNF/IL1/NF-κB-related programs. Module scores were compared across score-defined immune-aging-high spots, score-defined stromal-aging-high spots, psoriatic keratinocyte-enriched domains, and other spots. Representative spatial projections were generated for selected programs, including epithelial–mesenchymal transition, IFN gamma response, TNFα signaling via NFκB, immune-aging, stromal-aging, and global old-up scores.

For lesion-status comparisons, tissue sections rather than individual spatial spots were used as the statistical unit. Mean immune-aging score, overlap-removed immune-aging score, and inflammatory module scores were calculated for each section. Score-defined immune-aging-high region fraction was retained as a supporting threshold-based readout rather than the primary lesion-status measure. Section-level inflammatory module scores were calculated for IFN gamma response, cytokine/chemokine signaling, and antigen presentation. Spearman correlation analysis was used to assess section-level associations between immune-aging activity, overlap-removed immune-aging activity, high-region fraction, and inflammatory module activity. Patient identity was retained in paired visualizations when possible.

### 2.4 External spatial transcriptomic support analysis

The external support analysis was performed using GSE202011. The same aging-associated signatures, overlap-removed immune-aging score, and inflammatory modules were projected onto external spatial spots. Metadata columns were used to identify sample, patient, disease group, lesion status, and spatial coordinates. Disease and lesion status were harmonized into five groups: healthy, psoriasis non-lesional, psoriasis lesional, psoriatic arthritis non-lesional, and psoriatic arthritis lesional.

For each external section, mean immune-aging score, overlap-removed immune-aging score, mean stromal-aging score, global old-up score, IFN gamma response score, cytokine/chemokine score, and antigen-presentation score were calculated. Threshold-defined immune-aging-high spot fraction was also calculated as a supporting analysis using the discovery-cohort scoring framework. Section-level group comparisons and Spearman correlation analyses were used to examine whether immune-aging activity or immune-aging-high spot fraction was associated with inflammatory module activity across the external dataset. Representative external sections were visualized using histology reference images and spatial projections of immune-aging, overlap-removed immune-aging, IFN gamma response, and immune-aging-high spot classification.

### 2.5 Aging skin single-cell reference projection and spatial cellular-context enrichment

**Figure 1:**
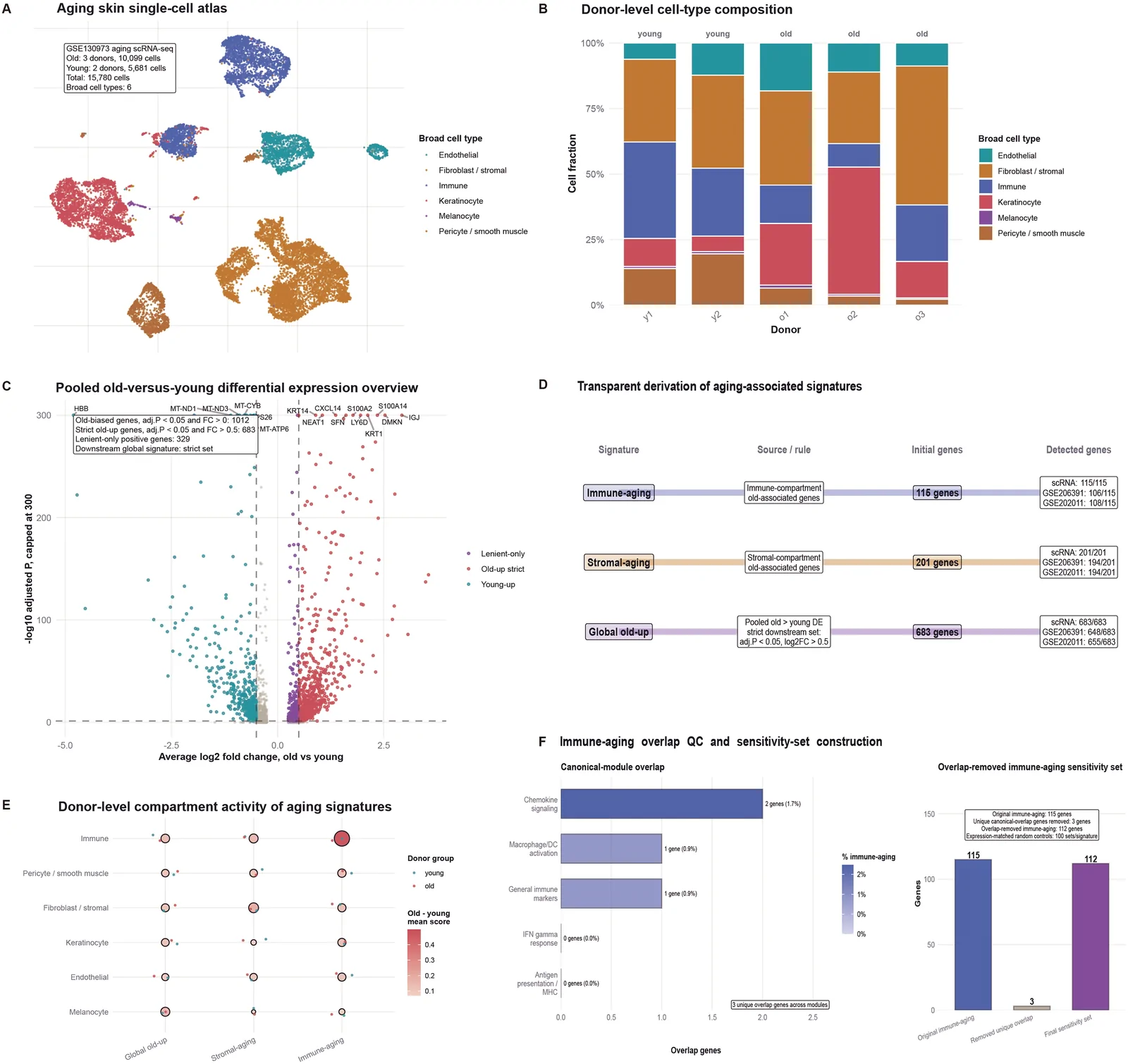
Single-cell analysis defines global, stromal, and immune-associated aging programs in skin. **(A)** UMAP visualization of the aging skin single-cell dataset, split by age group and broad cellular compartment. Young and old cells occupied shared broad skin compartments rather than forming completely separated age-specific clusters. **(B)** Donor-level broad cell-type composition across young and old skin samples. This analysis was used descriptively because the dataset contained two young and three old donors. **(C)** Definition of the old-biased gene pool and strict global old-up signature. The broader old-biased gene pool contained 1,012 genes with adjusted P < 0.05 and positive old-versus-young fold change. The strict global old-up signature contained 683 genes with adjusted P < 0.05 and average log2 fold change > 0.5. The 329 remaining genes were positive but did not meet the strict fold-change threshold. The strict 683-gene score closely tracked the broader 1,012-gene score at the donor-compartment level. **(D)** Summary of the three downstream aging-associated signatures and their detected gene counts across the aging scRNA-seq dataset, the discovery spatial dataset GSE206391, and the external spatial dataset GSE202011. **(E)** Donor-by-cell-type module-score activity for the global old-up, stromal-aging, and immune-aging signatures. Scores were used descriptively to summarize compartment-level activity. **(F)** Overlap between the immune-aging signature and canonical immune/inflammatory modules, together with expression-matched random gene-set controls. Unique canonical immune-overlap genes were CXCL2, CXCL3, and LYZ; no immune-aging genes overlapped with the IFN gamma response or antigen-presentation/MHC modules.

To provide broad cellular-compartment context for score-defined immune-aging-high spatial regions, immune-aging, global old-up, and inflammatory modules were projected onto the GSE130973 aging skin single-cell RNA-seq reference used for signature definition. This analysis used the obj_skin object analyzed in **Figure 1**, including young samples y1 and y2 and old samples o1, o2, and o3. Cells were grouped into annotated major cellular compartments, including keratinocytes, immune cells, fibroblast/stromal cells, endothelial cells, melanocytes, and pericyte/smooth muscle cells. For each cell population, mean module score and the fraction of cells above a predefined module-score threshold were calculated. This projection summarized module activity across broad aging-skin cellular compartments and was not used to infer spot-level cell-type proportions.

Marker-based enrichment analysis was performed in the discovery psoriasis spatial transcriptomic dataset GSE206391 to evaluate cellular-context programs enriched in score-defined immune-aging-high spots. Predefined marker programs included dendritic/APC, macrophage/monocyte, T cell, cytotoxic lymphocyte, keratinocyte, inflamed keratinocyte, B/plasma cell, endothelial, fibroblast, pericyte/VSMC, and related skin-associated marker sets. Enrichment odds ratios and P values were calculated to determine whether score-defined immune-aging-high spots were enriched for specific cellular-context programs.

### 2.6 Cell culture and treatment

Human foreskin fibroblasts HFF-1 (ATCC SCRC-1041) were maintained in complete DMEM at 37 °C with 5% CO_2_. Cells were treated under four conditions: control, IFNγ, DOXO, and DOXO+IFNγ. For the DOXO condition, cells were treated with 250 nM doxorubicin for 24 h followed by 5 days of recovery. For the IFNγ condition, cells were treated with 20 ng/mL IFNγ for 48 h. For the DOXO+IFNγ condition, cells were first treated with 250 nM doxorubicin for 24 h, allowed to recover for 5 days, and then treated with IFNγ for 48 h.

### 2.7 Staining

Senescence-associated β-galactosidase staining was performed using the Senescence β-Galactosidase Staining Kit (9860, Cell Signaling Technology) according to the manufacturer’s instructions. Representative brightfield images were acquired using a Zeiss inverted microscope.

Cells were fixed with 4% paraformaldehyde and processed for standard immunofluorescence staining. The following primary antibodies were used: rabbit anti-Lamin B1 (1:10000, Abcam ab16048), rabbit anti-γH2AX (1:1000, Cell Signaling Technology 9718S), rabbit anti-p15INK4b/CDKN2B (1:1000, Abcam ab53034), anti-HLA-ABC (Invitrogen MA5-11723) and Phalloidin (1:10000, Thermofisher A12379). Alexa Fluor 555 secondary antibody (1:500, Invitrogen) was used for rabbit primary antibodies. Phalloidin and DAPI were used to visualize F-actin and nuclei, respectively. Images were acquired using a Zeiss inverted fluorescence microscope. Fluorescence intensity for Lamin B1, CDKN2B, γH2AX, and HLA-ABC was quantified from representative fields using the same image-acquisition settings across treatment groups and summarized as relative mean fluorescence intensity.

### 2.8 qPCR analysis

RNA was extracted from treated cells, reverse-transcribed into cDNA, and analyzed by quantitative PCR using a QuantStudio real-time PCR system. ACTB was used as the internal control, and relative expression was calculated using the 2^-ΔCt method. The following primers or assays were used: B2M, TaqMan assay Hs00187842_m1; ACTB forward 5′-ACCACCTTCAACTCCATCATG-3′ and reverse 5′-CTCCTTCTGCATCCTGTCG-3′; CDKN2A forward 5′-CCCAACGCCCCGAACT-3′ and reverse 5′-GCAGAAGAGCTGCTACGTGAA-3′; CDKN2B forward 5′-GAATGCGCGAGGAGAACAA-3′ and reverse 5′-CATCATCATGACCTGGATCGC-3′; LMNB1 forward 5′-GGGAAGTTTATTCGCTTGAAGA-3′ and reverse 5′-ATCTCCCAGCCTCCCATT-3′; CD74 forward 5′-AAGCCTGTGAGCAAGATGCGCA-3′ and reverse 5′-AGCAGGTGCATCACATGGTCCT-3′; CXCL9 forward 5′-CTGTTCCTGCATCAGCACCAAC-3′ and reverse 5′-TGAACTCCATTCTTCAGTGTAGCA-3′.

### 2.9 Flow cytometry

Surface HLA-DR expression was measured by flow cytometry. Cells were harvested, stained with APC-conjugated anti-HLA-DR antibody (17-9956-42, Invitrogen; 5 µL/test), and analyzed on a BD flow cytometer. Propidium iodide was used for dead-cell exclusion. HLA-DR-positive fractions and APC geometric mean fluorescence intensity were quantified across three independent experiments.

### 2.10 Statistical analysis and visualization

Statistical analyses were performed in R and GraphPad Prism. For transcriptomic analyses, differential expression and enrichment analyses used false discovery rate correction where applicable. For spatial transcriptomic comparisons, tissue sections rather than individual spots were used as the statistical unit unless otherwise stated. Section-level associations were assessed using Spearman correlation. Because the discovery spatial cohort contained only three patients with multiple sections per patient, section-level statistics were treated as exploratory and were not used for patient-level effect-size estimation. For *in vitro* experiments, quantitative qPCR, immunofluorescence-intensity, and flow-cytometry readouts from the four treatment conditions were analyzed as a 2 × 2 factorial design, with DOXO treatment and IFNγ stimulation as the two factors. Two-way ANOVA was used to evaluate the main effects of DOXO and IFNγ and the DOXO × IFNγ interaction where applicable. Selected *post hoc* multiple comparisons were then performed, including direct comparisons between IFNγ and DOXO+IFNγ conditions to test whether IFNγ-responsive markers were retained after DOXO-induced senescence-like stress. Adjusted P values are reported for selected *post hoc* comparisons. Statistical significance was defined as P < 0.05. In figures, asterisks indicate adjusted P values for selected *post hoc* comparisons: *P < 0.05, **P < 0.01, ***P < 0.001, ****P < 0.0001; ns, not significant.

## 3 Results

### 3.1 Single-cell analysis defines compartment-resolved aging programs in skin

Aging can affect the skin through multiple biological compartments, including epithelial differentiation, stromal remodeling, vascular changes, and immune activation. Therefore, before asking whether aging-related programs are spatially organized in psoriatic tissue, we first defined aging-associated transcriptional programs in normal skin at single-cell resolution. We analyzed an aging skin scRNA-seq dataset containing 15,780 cells from five samples, including 5,681 cells from two young samples and 10,099 cells from three old samples. Young cells were derived from samples y1 and y2, containing 3,120 and 2,561 cells, respectively, whereas old cells were derived from samples o1, o2, and o3, containing 3,328, 2,222, and 4,549 cells, respectively. These data were used to define and quality-control aging-associated skin signatures before projection into psoriasis spatial transcriptomic datasets.

Cells were annotated into six major skin compartments based on canonical marker expression and visualized on a shared UMAP embedding (**Figure 1A**; **Supplementary Figures 1A, B**).Fibroblast/stromal cells represented the largest compartment, comprising 6,107 cells (38.7%), followed by immune cells (3,480 cells, 22.1%), keratinocytes (2,977 cells, 18.9%), endothelial cells (1,764 cells, 11.2%), pericyte/smooth muscle cells (1,329 cells, 8.4%), and melanocytes (123 cells, 0.8%). When young and old cells were projected onto the same embedding, both age groups occupied the same broad cellular compartments rather than forming completely separated age-specific clusters (**Figure 1A**). This suggested that aging in this dataset was not represented by the emergence of entirely new major cell populations, but instead by compositional changes and transcriptional remodeling within shared skin compartments.

We next summarized broad cell-type composition at the donor level (**Figure 1B**). Because this dataset contained only two young and three old donors, this analysis was used descriptively rather than as a formal statistical test of age-associated compositional differences. Keratinocytes increased from 8.5% of young cells to 24.7% of old cells, corresponding to a 16.2 percentage-point increase. Fibroblast/stromal cells increased from 33.3% to 41.7%, and endothelial cells increased from 9.0% to 12.4%. In contrast, immune cells decreased from 31.9% in young cells to 16.5% in old cells, and pericyte/smooth muscle cells decreased from 16.5% to 3.9%. Melanocytes remained rare in both groups, accounting for 0.83% of young cells and 0.75% of old cells. These donor-level patterns showed that broad compartment composition varied across age groups in this dataset. At the same time, they also raised an important analytical issue: pooled old-versus-young differential expression could reflect both transcriptional changes and shifts in cell-type abundance. We therefore used pooled differential expression as an initial overview of aging-associated directionality and then interpreted these results together with cell-type-level scoring.

Pooled old-versus-young differential expression identified a broad aging-associated transcriptional shift (**Figure 1C**). Using adjusted P < 0.05 as the significance cutoff, 1,012 genes showed positive old-versus-young fold change and were considered part of a broader old-biased gene pool. Among these, 683 genes further met the stricter threshold of adjusted P < 0.05 and average log2 fold change > 0.5 and were defined as the strict global old-up signature used for downstream analysis. The remaining 329 genes showed positive old-versus-young fold change but did not pass the strict fold-change threshold. Using the same strict fold-change threshold in the opposite direction, 408 genes were identified as young-upregulated genes. The strict old-up genes included epidermal differentiation and stress-associated markers such as KRT1, KRT14, SFN, S100A2, S100A14, LY6D, and DMKN, pointing to epithelial differentiation, barrier-related stress, and inflammatory remodeling as major components of the old-skin transcriptional profile. Young-upregulated genes included UQCRB, MT-ND1, CXCL14, HBB, and NEAT1. The strict 683-gene score closely tracked the broader 1,012-gene old-biased score at the donor-compartment level, supporting the use of the stricter gene set for downstream spatial projection (**Supplementary Figure 1C**). Because this analysis was performed across pooled cells, we treated it as a global aging overview rather than as definitive evidence that each gene represented cell-intrinsic aging within a particular compartment.

We therefore summarized the aging analysis into three signatures for downstream spatial projection (**Figure 1D**). The downstream global old-up signature contained the 683 strict old-up genes and was associated with keratinization, epidermal stress, and inflammatory remodeling. The stromal-aging signature contained 201 stromal-compartment old-associated genes and was associated with extracellular matrix organization, collagen biology, and matrix remodeling. The immune-aging signature contained 115 immune-compartment old-associated genes and was associated with immune activation-related features. Most signature genes were detected in both spatial transcriptomic datasets used later in the study. In the discovery spatial dataset GSE206391, 648 of 683 global old-up genes, 194 of 201 stromal-aging genes, and 106 of 115 immune-aging genes were detected. In the external spatial dataset GSE202011, 655 of 683 global old-up genes, 194 of 201 stromal-aging genes, and 108 of 115 immune-aging genes were detected (**Figure 1D**; **Supplementary Figure 1D**; **Supplementary Tables 1**–**3**).

After defining these three signatures, we summarized their module-score activity at the donor-by-cell-type level (**Figure 1E**). The global old-up program showed higher activity in several old-donor compartments, including melanocyte, immune, and fibroblast/stromal compartments. The stromal-aging program was most evident in fibroblast/stromal and pericyte/smooth muscle compartments, whereas the immune-aging program showed the strongest activity in immune cells, with additional signal in pericyte/smooth muscle and fibroblast/stromal compartments. These donor-level patterns were used descriptively and were not treated as cell-level statistical inference. Together, they supported the separation of aging-associated skin programs into a broad old-up program, a stromal-remodeling program, and an immune-associated program.

Because the immune-aging signature included immune-associated genes, we explicitly quantified its overlap with canonical inflammatory, IFN-response, antigen-presentation, and immune-cell marker modules. The 115-gene immune-aging signature showed limited overlap with these canonical modules. It overlapped with two chemokine signaling genes, one macrophage/DC activation gene, and one general immune marker gene. Because LYZ overlapped with both the macrophage/DC activation and general immune marker categories, the total number of unique canonical immune-overlap genes was three: CXCL2, CXCL3, and LYZ. No immune-aging genes overlapped with the IFN gamma response or antigen-presentation/MHC modules (**Figure 1F**; **Supplementary Figure 1E**; **Supplementary Table 3**). We therefore generated an overlap-removed immune-aging sensitivity set by removing these three unique canonical immune-overlap genes, leaving 112 genes for downstream sensitivity analysis (**Figure 1G**). Expression-matched random gene-set controls further supported that the observed aging-associated signature activities were not reproduced by random gene sets of comparable size and expression level (**Supplementary Figure 1F**).

### 3.2 Aging-associated immune and stromal programs show spatial organization within psoriatic tissue domains

**Figure 2:**
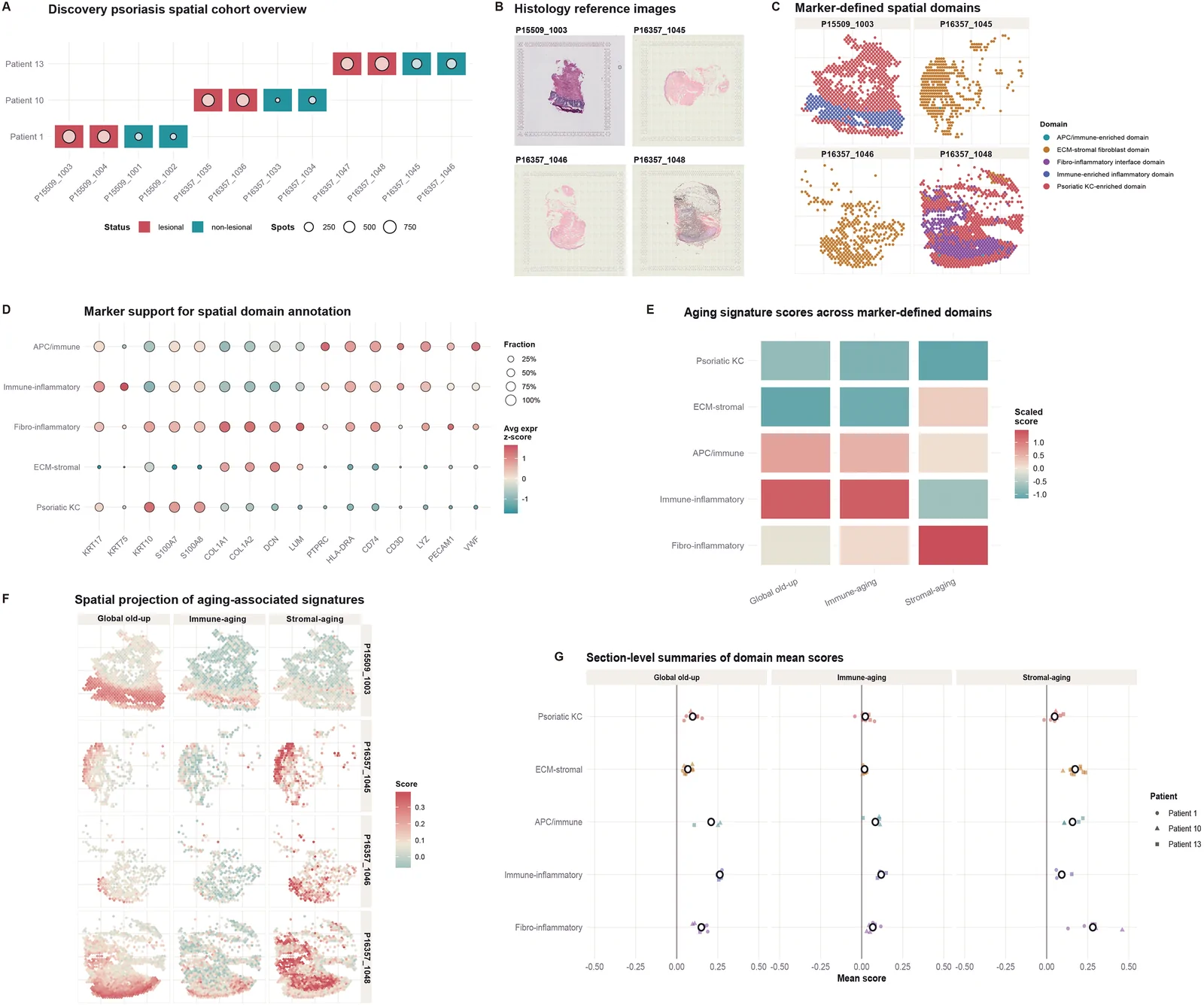
Projection of aging-associated signatures across marker-defined spatial domains in the discovery psoriasis spatial cohort. **(A)** Overview of the discovery psoriasis spatial transcriptomic cohort. The cohort included 12 tissue sections from three patients, with lesional and non-lesional sections and 9,830 retained spots. **(B)** Representative histology reference images. Histology images were used as morphological references and not for quantitative image-registration-based analysis. **(C)** Marker-defined spatial domains in representative sections. Domain labels indicate spot-level transcriptional states rather than purified cell populations. **(D)** Marker support for spatial domain annotation. Dot size indicates the fraction of spots expressing each marker, and color indicates average expression z-score. **(E)** Aging-associated signature scores across marker-defined spatial domains. Scores are shown for the strict 683-gene global old-up signature, the 115-gene immune-aging signature, and the 201-gene stromal-aging signature. **(F)** Spatial projection of aging-associated signatures in representative sections. **(G)** Section-level summaries of domain mean scores. Each point represents a section-level domain mean, and point shape indicates patient identity.

We next projected the three aging-associated signatures defined above onto a discovery psoriasis spatial transcriptomic cohort to examine how these programs were distributed within diseased skin. This cohort included 12 tissue sections from three psoriasis patients, with six lesional and six non-lesional sections and 9,830 retained spots in total (**Figure 2A**). Lesional sections contributed 6,077 spots, whereas non-lesional sections contributed 3,753 spots. Histology images from representative sections were used as morphological references for the spatial transcriptomic profiles, but not for quantitative image-registration-based analysis (**Figure 2B**).

We first annotated the major spatial domains represented in the dataset using epithelial, stromal, immune, vascular, and inflammatory marker programs. The marker-based annotation identified five major tissue-associated transcriptional states: psoriatic keratinocyte-enriched, ECM-stromal fibroblast, APC/immune-enriched, immune-enriched inflammatory, and fibro-inflammatory interface domains (**Figure 2C**; **Supplementary Figures 2A, B**). These domain labels refer to spot-level transcriptional states rather than purified cell populations, because each Visium spot may contain heterogeneous cellular mixtures.

Marker expression supported the domain assignments (**Figure 2D**). The psoriatic keratinocyte-enriched domain showed epithelial and psoriasis-associated markers, including KRT10, S100A7, and S100A8. The ECM-stromal domain was marked by matrix genes such as COL1A1, COL1A2, DCN, and LUM. APC/immune-enriched and immune-inflammatory domains expressed immune and antigen-presentation-associated markers, including HLA-DRA, CD74, CD3D, LYZ, and PTPRC. The fibro-inflammatory interface domain showed a mixed stromal and inflammatory profile. The distribution of these domains also tracked lesion status: the psoriatic keratinocyte-enriched domain was almost entirely confined to lesional tissue, accounting for 3,240 lesional spots but only four non-lesional spots, whereas stromal-associated domains were more prominent outside the lesional keratinocyte-dominant areas.

We then projected the strict 683-gene global old-up, 115-gene immune-aging, and 201-gene stromal-aging signatures onto these marker-defined domains. Domain-level scoring showed that the aging-associated programs were not evenly distributed across psoriatic tissue (**Figure 2E**). Global old-up and immune-aging scores were higher in immune-enriched inflammatory and APC/immune-enriched domains, whereas stromal-aging was highest in fibro-inflammatory and ECM-stromal domains. The psoriatic keratinocyte-enriched domain, despite being the dominant lesional domain, showed lower immune-aging and stromal-aging scores than the immune-associated and stromal-interface domains.

Spatial projection of the three signatures showed the same domain-associated pattern within representative sections (**Figure 2F**), and all-section projections showed that these patterns were not restricted to selected examples (**Supplementary Figure 2C**). Global old-up and immune-aging scores were higher in immune-associated regions, whereas stromal-aging scores localized more strongly to stromal and fibro-inflammatory areas. These patterns were observed across multiple representative lesional and non-lesional sections rather than being restricted to a single sample. Section-level summaries of domain mean scores further supported the separation between immune-associated and stromal-associated aging programs while retaining patient identity in the visualization (**Figure 2G**). Across sections, APC/immune and immune-inflammatory domains showed higher global old-up and immune-aging scores, whereas fibro-inflammatory and ECM-stromal domains showed higher stromal-aging scores. Because Visium spots contain mixed cellular populations, we next added sensitivity analyses to evaluate whether the projected immune-aging pattern was explained solely by canonical immune-overlap genes or local immune/APC marker abundance. First, we removed the three unique canonical immune-overlap genes identified in **Figure 1**, CXCL2, CXCL3, and LYZ, and recalculated an overlap-removed immune-aging score. The original and overlap-removed immune-aging scores remained highly correlated at the domain level, and representative spatial projections showed similar patterns (**Supplementary Figures 2D, E**). Second, we calculated marker-based cellular-context scores for keratinocyte, fibroblast/stromal, APC/myeloid, T cell, NK/cytotoxic, B/plasma cell, endothelial, and pericyte/VSMC programs across marker-defined domains (**Supplementary Figure 2F**). We then generated an immune/APC marker-adjusted immune-aging residual by regressing the immune-aging score against immune/APC-related marker programs. The adjusted residual retained a domain-associated spatial pattern and remained correlated with the original immune-aging domain-level signal (**Supplementary Figures 2G, H**). These sensitivity analyses do not assign a single cell type of origin to each Visium spot, but they reduce the likelihood that the projected immune-aging pattern is explained solely by a few overlapping immune genes or by local immune/APC marker-program abundance.

Together, these analyses showed that aging-associated signatures from **Figure 1** mapped to specific marker-defined spatial domains in psoriatic tissue rather than appearing as a uniform lesion-wide signal. The global old-up and immune-aging programs were preferentially associated with APC/immune and immune-inflammatory domains, whereas the stromal-aging program was associated with fibro-inflammatory and stromal-remodeling domains. Sensitivity analyses using overlap-removed and immune/APC marker-adjusted scores supported that the projected immune-aging pattern was not driven only by a small number of canonical immune-overlap genes or by local immune/APC marker-program abundance. This domain-level organization raised the next question of whether high-scoring immune-associated and stromal-associated regions have distinct molecular programs.

### 3.3 Score-defined aging-high spatial regions show distinct stromal-remodeling and immune-associated programs

**Figure 3:**
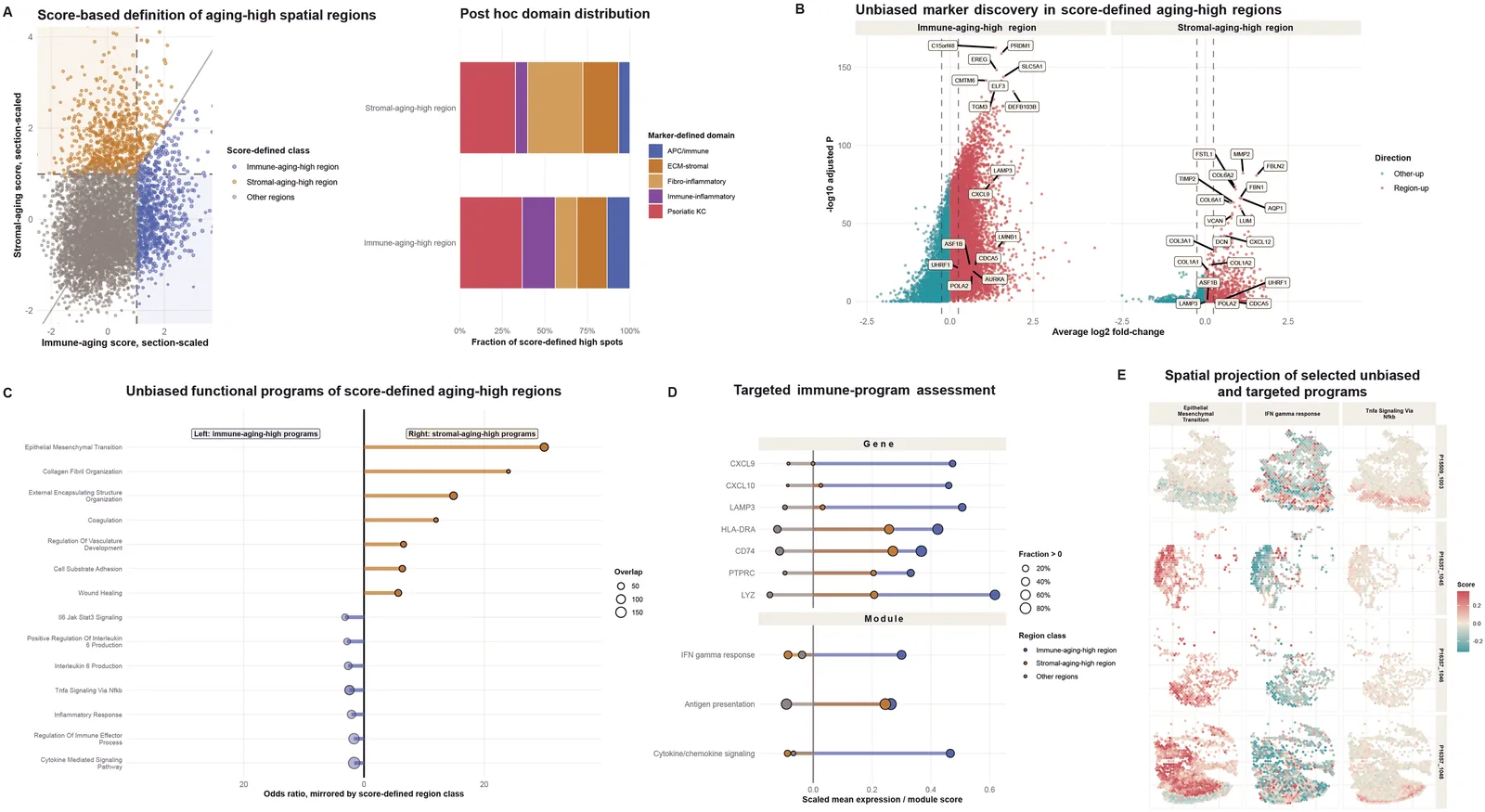
Score-defined aging-high spatial regions show distinct stromal-remodeling and immune-associated programs. **(A)** Score-based definition of aging-high spatial regions. Individual Visium spots were classified using section-scaled immune-aging and stromal-aging scores. Marker-defined domains were overlaid *post hoc* to summarize the domain distribution of score-defined immune-aging-high and stromal-aging-high regions. **(B)** Unbiased marker discovery in score-defined aging-high regions. Volcano plots show genes increased in immune-aging-high or stromal-aging-high regions compared with other regions. **(C)** Unbiased functional programs of score-defined aging-high regions. Enriched pathways were summarized separately for immune-aging-high and stromal-aging-high regions. Odds ratios are mirrored by score-defined region class. **(D)** Targeted immune-program assessment across immune-aging-high, stromal-aging-high, and other regions. Dot size indicates the fraction of spots with detectable expression or positive module score, and color indicates region class. **(E)** Spatial projection of selected unbiased and targeted programs in representative sections.

The domain-level projection in **Figure 2** suggested that immune-aging and stromal-aging scores were spatially organized across psoriatic tissue. To avoid defining aging-high regions solely by marker-defined domain labels, we next classified individual Visium spots using their section-scaled immune-aging and stromal-aging scores. Spots with high immune-aging activity were assigned to an immune-aging-high region class, whereas spots with high stromal-aging activity were assigned to a stromal-aging-high region class; the remaining spots were retained as other regions (**Figure 3A**). Marker-defined domains were then overlaid *post hoc* to summarize where these score-defined high regions were located. This analysis showed that immune-aging-high regions were enriched in immune-associated domains but were not identical to any single marker-defined domain, whereas stromal-aging-high regions were enriched in stromal and fibro-inflammatory contexts. Threshold-sensitivity analysis using top 10%, 15%, and 20% cutoffs showed similar *post hoc* domain distributions, supporting the robustness of the score-defined high-region classification (**Supplementary Figure 3A**).

We then performed unbiased marker discovery to determine which genes distinguished the score-defined immune-aging-high and stromal-aging-high regions (**Figure 3B**). Immune-aging-high regions showed increased expression of immune- and inflammation-associated genes, including CXCL9 and LAMP3, together with selected stress- or proliferation-associated genes such as LMNB1, AURKA, UHRF1, and POLA2. Stromal-aging-high regions were marked by extracellular matrix and stromal-remodeling genes, including DCN, COL1A2, COL6A1, COL6A2, FBN1, VCAN, and FSTL1. Thus, the two score-defined aging-high region classes were separated not only by their aging-score profiles, but also by distinct marker-gene patterns.

Pathway enrichment analysis further supported this separation (**Figure 3C**). Immune-aging-high regions were enriched for inflammatory and cytokine-linked programs, including IL6/JAK/STAT3 signaling, regulation of IL6 production, TNFα signaling via NFκB, inflammatory response, regulation of immune effector processes, and cytokine-mediated signaling pathways. In contrast, stromal-aging-high regions were enriched for extracellular matrix and tissue-remodeling programs, including epithelial–mesenchymal transition, collagen fibril organization, external encapsulating structure organization, regulation of vasculature development, cell–substrate adhesion, and wound healing. These enrichment results matched the marker-gene patterns and separated immune-aging-high regions from stromal-aging-high regions at the pathway level.

Because the unbiased analysis highlighted inflammatory and cytokine-linked features in the immune-aging-high regions, we next evaluated targeted immune and inflammatory modules directly (**Figure 3D**). Immune-aging-high regions showed higher CXCL9, CXCL10, LAMP3, HLA-DRA, CD74, PTPRC, and LYZ expression compared with stromal-aging-high and other spatial regions. The same region class also showed higher IFN gamma response and cytokine/chemokine signaling module scores. Antigen-presentation activity was present in more than one region class, with signal in both immune-associated and stromal-associated contexts. These results indicated that the immune-aging-high regions carried IFN-linked chemokine and immune-activation features, rather than representing a purely score-defined artifact.

Spatial projection of representative programs showed that these molecular signatures occupied structured regions within individual tissue sections (**Figure 3E**). Epithelial–mesenchymal transition was strongest in stromal-associated regions, whereas IFN gamma response and TNFα signaling via NFκB showed spatially structured inflammatory patterns that overlapped with immune-associated tissue areas. All-section maps of the score-defined region classes further showed that immune-aging-high and stromal-aging-high regions were distributed across multiple sections rather than being restricted to a single tissue fragment (**Supplementary Figure 3D**).

We also performed sensitivity analyses to test whether the score-defined high-region assignments were dependent on cutoff choice or on the small number of canonical immune-overlap genes. Top 10%, 15%, and 20% score cutoffs produced similar *post hoc* domain distributions of immune-aging-high and stromal-aging-high regions (**Supplementary Figure 3A**). Re-defining immune-aging-high regions using the 112-gene overlap-removed immune-aging score produced highly concordant high-region assignments with the original immune-aging score (Jaccard index = 0.876; **Supplementary Figure 3B**). In addition, within-domain high-versus-low comparisons showed that immune-aging and inflammatory program gradients were retained within immune-associated domains, whereas stromal-aging gradients were retained within stromal-associated domains (**Supplementary Figure 3C**). These analyses support that the score-defined aging-high regions were not solely an artifact of marker-defined domain labels or a single arbitrary cutoff.

Together, these analyses showed that score-defined aging-high regions were not equivalent tissue states. Immune-aging-high regions carried IFN-linked chemokine, inflammatory, and immune-activation features, together with selected stress- or proliferation-associated markers, whereas stromal-aging-high regions were dominated by extracellular matrix organization, epithelial–mesenchymal transition, collagen biology, and stromal remodeling. Because the immune-aging-high region class contained both aging-associated and inflammatory features, we next asked whether immune-aging activity was increased in lesional psoriasis and whether it was associated with section-level inflammatory activity.

### 3.4 Immune-aging activity is increased in lesional psoriasis and tracks IFN-associated inflammatory activity

After defining score-defined aging-high spatial regions, we next asked whether immune-aging activity was increased in lesional psoriasis and whether it was associated with inflammatory tissue activity. Because threshold-defined high-region fraction summarizes a continuous spatial score using a discrete cutoff, section-level immune-aging activity was used as the primary readout, while score-defined high-region fraction was retained as a supporting analysis. Tissue sections, rather than individual Visium spots, were used as the analytical unit, and patient identity was retained in the visualization.

**Figure 4:**
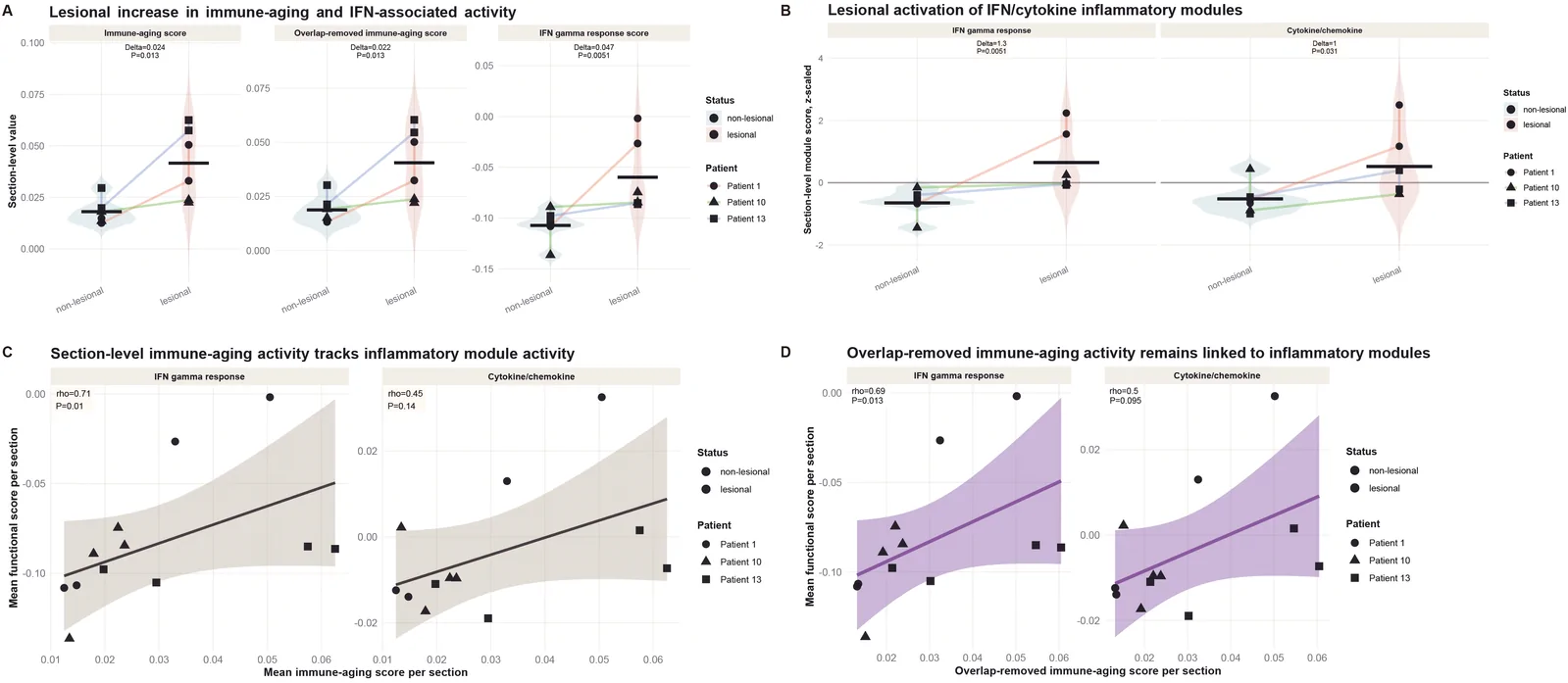
Lesional increase in immune-aging and IFN-associated inflammatory activity. **(A)** Section-level comparison of immune-aging score, overlap-removed immune-aging score, and IFN gamma response score between non-lesional and lesional sections. Lines connect sections from the same patient. **(B)** Lesional activation of IFN/cytokine inflammatory modules. Section-level z-scaled module scores are shown for IFN gamma response and cytokine/chemokine activity. **(C)** Section-level correlations between mean immune-aging score and inflammatory module activity. **(D)** Section-level correlations between overlap-removed immune-aging score and inflammatory module activity. The overlap-removed immune-aging score excludes the three canonical immune-overlap genes CXCL2, CXCL3, and LYZ.

Lesional sections showed increased immune-aging activity compared with non-lesional sections (**Figure 4A**). The mean immune-aging score increased in lesional tissue (Δ = 0.024, exploratory section-level 95% CI 0.0073–0.035, P = 0.013). This increase was retained when using the overlap-removed immune-aging score, which excludes the three canonical immune-overlap genes CXCL2, CXCL3, and LYZ (Δ = 0.022, exploratory section-level 95% CI 0.0058–0.032, P = 0.013). IFN gamma response activity was also higher in lesional sections (Δ = 0.047, exploratory section-level 95% CI 0.016–0.093, P = 0.0051). Thus, lesional tissue showed concordant increases in immune-aging activity, overlap-removed immune-aging activity, and IFN-associated inflammatory activity.

We then evaluated inflammatory module activity by lesion status. IFN gamma response and cytokine/chemokine modules were both increased in lesional sections (**Figure 4B**). The IFN gamma response module increased by 1.3 in lesional tissue compared with non-lesional tissue (exploratory section-level 95% CI 0.43–2.6, P = 0.0051), and the cytokine/chemokine module increased by 1.0 (exploratory section-level 95% CI 0.14–2.4, P = 0.031). These results indicate that lesional sections showed increased IFN- and chemokine-associated inflammatory activity.

Section-level correlation analysis further linked immune-aging activity with inflammatory module activity (**Figure 4C**). Mean immune-aging score per section was positively correlated with IFN gamma response activity (rho = 0.71, P = 0.01). The association with cytokine/chemokine activity showed the same positive direction but was weaker in this section-level cohort (rho = 0.45, P = 0.14). These results suggest that immune-aging activity tracked IFN-associated inflammatory activity more strongly than broader cytokine/chemokine activity.

We next repeated the correlation analysis using the overlap-removed immune-aging score (**Figure 4D**). The overlap-removed immune-aging score remained positively correlated with IFN gamma response activity (rho = 0.69, P = 0.013). Its association with cytokine/chemokine activity was positive but weaker (rho = 0.50, P = 0.095). The preservation of the IFN-associated relationship after overlap removal provided a sensitivity analysis for the section-level immune-aging signal.

We also evaluated score-defined high-region fraction as a supporting threshold-based readout. This fraction showed only a weak, non-significant lesional shift (Δ = 0.0012, P = 0.13; **Supplementary Figure 4A**). Region-fraction analysis showed a positive association with cytokine/chemokine activity (rho = 0.84, P = 0.00064), whereas the association with IFN gamma response was weaker (**Supplementary Figure 4B**). Additional exploratory tissue-level analyses with psoriatic keratinocyte programs and patient-level paired summaries are shown in **Supplementary Figures 4C, D**.

Together, these section-level analyses show that continuous immune-aging activity, rather than threshold-defined high-region abundance alone, was increased in lesional psoriasis in this discovery cohort and was associated with IFN-linked inflammatory activity. The overlap-removed immune-aging score showed the same direction of change and a similar IFN association, providing a sensitivity analysis for the module-level observation. These results support tissue-level co-organization between immune-aging activity and lesional inflammatory architecture, while the spatial data remain observational.

### 3.5 External spatial cohort provides supportive evidence for immune-aging activity and inflammatory coupling

**Figure 5:**
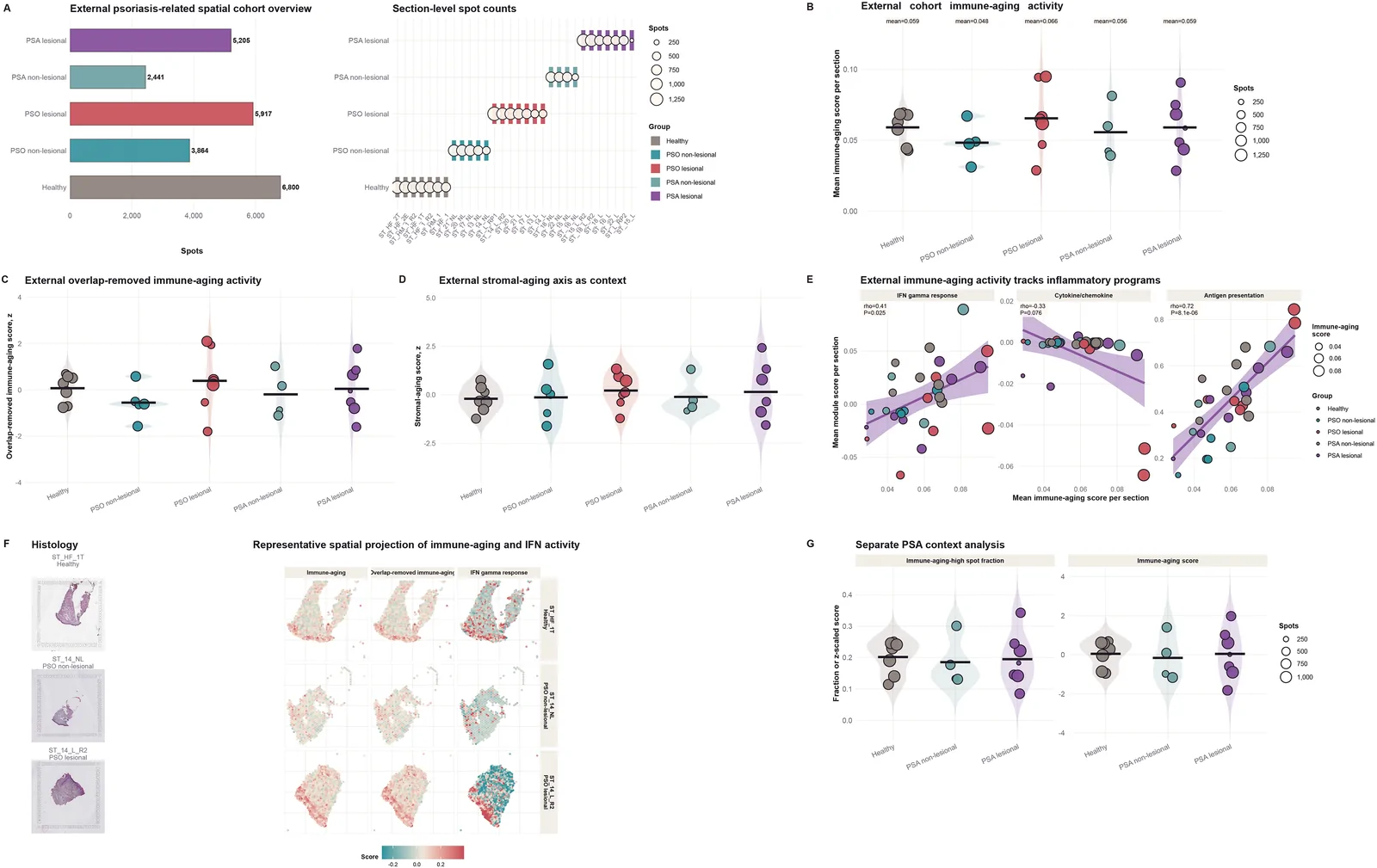
External spatial cohort provides supportive evidence for immune-aging activity and inflammatory coupling. **(A)** Overview of the external psoriasis-related spatial transcriptomic cohort. The cohort included healthy skin, psoriasis non-lesional, psoriasis lesional, psoriatic arthritis non-lesional, and psoriatic arthritis lesional sections. Left, total retained spot counts by group. Right, section-level spot counts, with point size indicating the number of retained spots per section. **(B)** Section-level immune-aging activity across external cohort groups. Each point represents one tissue section, and point size indicates retained spot count. Horizontal bars indicate group means. **(C)** Section-level overlap-removed immune-aging activity across external cohort groups. The overlap-removed immune-aging score was calculated after removing the three canonical immune-overlap genes CXCL2, CXCL3, and LYZ from the original immune-aging signature. **(D)** Section-level stromal-aging activity across external cohort groups, shown as a context axis for broader tissue remodeling. **(E)** Section-level correlations between mean immune-aging score and inflammatory module activity across the external cohort. Correlations are shown for IFN gamma response, cytokine/chemokine activity, and antigen-presentation activity. Point color indicates cohort group, and point size indicates immune-aging score. **(F)** Representative external spatial sections showing histology reference images and spatial projection of immune-aging, overlap-removed immune-aging, and IFN gamma response scores. Histology images were used as morphological references, not for quantitative image-registration-based analysis. **(G)** Separate psoriatic arthritis context analysis showing immune-aging-high spot fraction and mean immune-aging score across healthy, psoriatic arthritis non-lesional, and psoriatic arthritis lesional sections.

We next tested whether the immune-aging activity and inflammatory coupling observed in the discovery cohort could be detected in an independent spatial transcriptomic dataset. The same scoring framework and discovery-cohort threshold were applied to GSE202011, an external cohort containing healthy skin, cutaneous psoriasis, and psoriatic arthritis skin sections. This dataset included 24,227 spatial spots from 30 tissue sections across 9 individuals (**Figure 5A**). The cohort contained 6,800 healthy spots from 7 sections and 3 individuals, 3,864 psoriasis non-lesional spots from 5 sections and 5 individuals, 5,917 psoriasis lesional spots from 7 sections and 6 individuals, 2,441 psoriatic arthritis non-lesional spots from 4 sections and 4 individuals, and 5,205 psoriatic arthritis lesional spots from 7 sections and 5 individuals. In this external cohort, we projected the immune-aging, overlap-removed immune-aging, stromal-aging, and inflammatory module scores at the section level, while retaining immune-aging-high spot fraction as a supporting threshold-based analysis.

At the section level, immune-aging activity showed a modest increase in psoriasis lesional sections compared with psoriasis non-lesional sections and healthy controls, although group distributions overlapped across the external cohort (**Figure 5B**). The overlap-removed immune-aging score showed a similar pattern (**Figure 5C**). Because the external cohort contained healthy, cutaneous psoriasis, and psoriatic arthritis sections, these group-level patterns were interpreted in relation to broader tissue context rather than as a lesion-specific binary marker.

We next asked whether section-level immune-aging activity was associated with inflammatory modules in the external cohort (**Figure 5E**). Across all 30 external sections, mean immune-aging score correlated positively with IFN gamma response activity (rho = 0.41, P = 0.025) and more strongly with antigen-presentation activity (rho = 0.72, P = 8.1 × 10^-6). In contrast, cytokine/chemokine activity showed an inverse trend that did not reach conventional significance (rho = -0.33, P = 0.076). Thus, the external cohort reproduced the IFN- and antigen-presentation-linked components of immune-aging activity more clearly than the broader cytokine/chemokine pattern.

Supporting analyses of immune-aging-high spot fraction, healthy/psoriasis-only sections, and psoriatic arthritis sections are shown in **Supplementary Figure 5**. These analyses indicated that threshold-based high-spot abundance was less consistent than continuous section-level immune-aging activity across the external cohort.

Representative external sections illustrated how immune-aging, overlap-removed immune-aging, and IFN gamma response scores were spatially organized within tissue (**Figure 5F**). A healthy reference section, a psoriasis non-lesional section, and a psoriasis lesional section were shown to visualize the spatial distribution of these programs. These representative maps supported the section-level analysis by showing that immune-aging and IFN-associated activity formed regionally organized patterns within external tissue sections.

Overall, the external cohort supported selected features of the discovery analysis. Section-level immune-aging activity was higher in a subset of psoriasis lesional sections, and the overlap-removed immune-aging score showed a similar pattern. Across the full external cohort, immune-aging activity was positively associated with IFN gamma response and most strongly associated with antigen-presentation activity. At the same time, the cytokine/chemokine association differed from the discovery cohort, and group distributions overlapped across healthy, psoriasis, and psoriatic arthritis sections. These results indicate that the most consistent external component was the IFN- and antigen-presentation-linked aspect of immune-aging-associated spatial organization.

### 3.6 Score-defined immune-aging-high regions are embedded in multicellular inflammatory contexts

**Figure 6:**
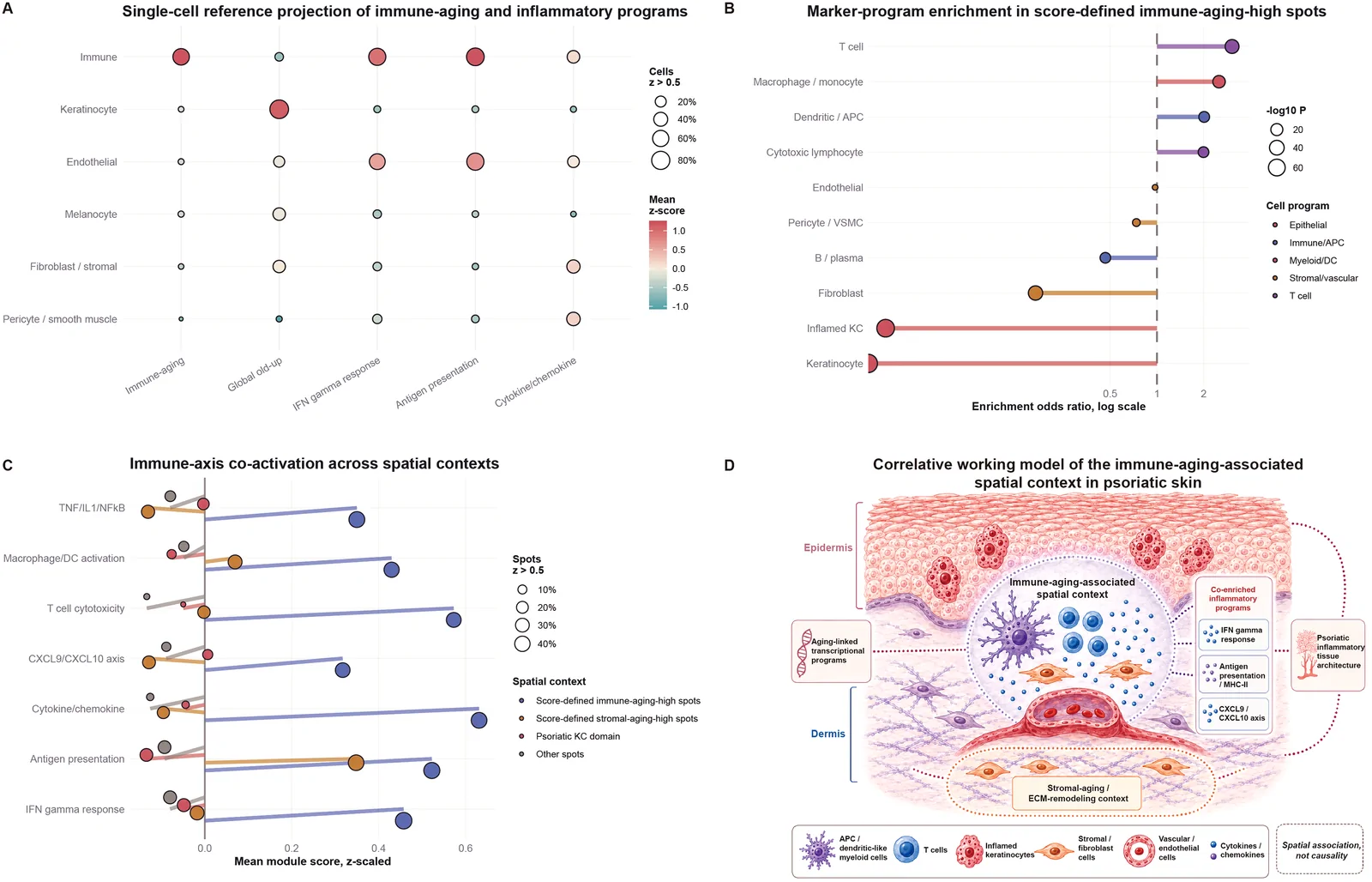
Score-defined immune-aging-high regions are embedded in multicellular inflammatory contexts. **(A)** Projection of immune-aging, global old-up, and inflammatory module scores onto the GSE130973 aging skin single-cell RNA-seq reference used for signature definition. This panel was generated from the obj_skin object analyzed in **Figure 1**, including young samples y1/y2 and old samples o1/o2/o3. Dot color indicates mean z-scaled module activity, and dot size indicates the fraction of cells above the indicated module-score threshold. **(B)** Marker-program enrichment in score-defined immune-aging-high spots from the discovery psoriasis spatial transcriptomic dataset GSE206391. **(C)** Immune-axis module activity across score-defined immune-aging-high spots, score-defined stromal-aging-high spots, psoriatic keratinocyte domains, and other spots in GSE206391. **(D)** Correlative working model of the immune-aging-associated spatial context in psoriatic skin. The schematic summarizes spatial co-enrichment among aging-associated transcriptional programs, immune-associated cellular context, IFN gamma response, antigen presentation/MHC-II activity, CXCL9/CXCL10 chemokine signaling, psoriatic inflammatory tissue architecture, and a related stromal-aging/ECM-remodeling context. The model is intended to summarize association and co-enrichment patterns and does not imply causal direction.

The preceding analyses showed that immune-aging activity was increased in lesional tissue and associated with IFN-linked and antigen-presentation programs. We next asked whether this spatial state could be attributed to a single cellular compartment, or whether it reflected a broader multicellular inflammatory context. To provide cellular context, immune-aging, global old-up, and inflammatory programs were projected onto the GSE130973 aging skin single-cell RNA-seq reference used to define the aging-associated signatures (**Figure 6A**). This projection summarized the compartmental distribution of each module in the aging skin reference and was not used to infer cell-type proportions for individual Visium spots.

In this aging skin single-cell reference, the immune-aging program was most strongly represented in immune cells, whereas the global old-up program was most prominent in keratinocytes (**Figure 6A**). IFN gamma response and antigen-presentation programs showed broader immune-lineage enrichment, with high activity in immune cells and detectable signal in endothelial and stromal compartments. Cytokine/chemokine activity was more broadly distributed across immune, fibroblast/stromal, and pericyte/smooth muscle populations. These patterns suggested that the immune-aging-associated spatial signal was unlikely to reflect a single isolated cell compartment alone and instead was consistent with a mixed tissue context involving immune, epithelial, stromal, and vascular-associated programs.

We then examined whether score-defined immune-aging-high spots were enriched for marker programs corresponding to specific cellular contexts in the spatial data (**Figure 6B**). Marker-based enrichment showed that immune-aging-high spots were enriched for immune-associated cellular programs, including T-cell, cytotoxic-lymphocyte, macrophage/monocyte, and dendritic/APC programs. Keratinocyte and inflamed-keratinocyte programs were not selectively enriched in immune-aging-high spots. This enrichment profile separated immune-aging-high spots from psoriatic keratinocyte-dominant regions and emphasized a broader immune-cell-enriched spatial context.

We next compared immune-axis module activity across score-defined immune-aging-high spots, score-defined stromal-aging-high spots, psoriatic keratinocyte domains, and other spots (**Figure 6C**). Score-defined immune-aging-high spots showed higher activity of several inflammatory programs, including IFN gamma response, CXCL9/CXCL10 chemokine axis, cytokine/chemokine signaling, antigen presentation, T-cell cytotoxicity, macrophage/DC activation, and TNF/IL1/NF-κB-related programs. Stromal-aging-high spots showed weaker immune-axis activity and were more consistent with a stromal remodeling context, whereas psoriatic keratinocyte domains showed a more restricted epithelial inflammatory profile. Thus, the immune-aging-high spatial state was accompanied by co-activation of multiple immune-associated programs within a spatially organized tissue context.

Based on these findings, we summarized the results in a correlative working model of the immune-aging-associated spatial context in psoriatic skin (**Figure 6D**). In this model, aging-associated transcriptional programs, immune-associated cellular context, IFN gamma response, antigen presentation/MHC-II activity, CXCL9/CXCL10 chemokine signaling, and psoriatic inflammatory tissue architecture are shown as spatially co-enriched features rather than as a causal pathway. Stromal-aging and ECM-remodeling programs are shown as a related but distinct remodeling context. This schematic is intended to summarize spatial co-enrichment patterns observed in the transcriptomic data and does not assign causal direction between aging programs, immune activation, and psoriatic tissue remodeling.

### 3.7 Senescence-like fibroblasts retain IFNγ-responsive antigen-presentation and chemokine programs

The spatial analyses above identified two related but distinct features of psoriatic tissue organization. Stromal-aging programs were associated with fibro-inflammatory and ECM-remodeling regions, whereas immune-aging activity was associated with IFN response, chemokine activity, antigen-presentation programs, and an immune-cell-enriched spatial context. These findings raised a narrower experimental question: whether stromal cells with senescence-associated features can retain responsiveness to inflammatory cytokine stimulation. To address this, we used human foreskin fibroblasts as a simplified stromal-cell system. Cells were treated with IFNγ for 2 days, DOXO for 1 day followed by 5 days of recovery, or DOXO for 1 day followed by 5 days of recovery and then IFNγ for 2 days. DOXO was used to induce a senescence-like DNA damage-associated state, whereas IFNγ was used to test whether antigen-presentation-associated and chemokine responses could still be induced in this stressed stromal-cell context.

**Figure 7:**
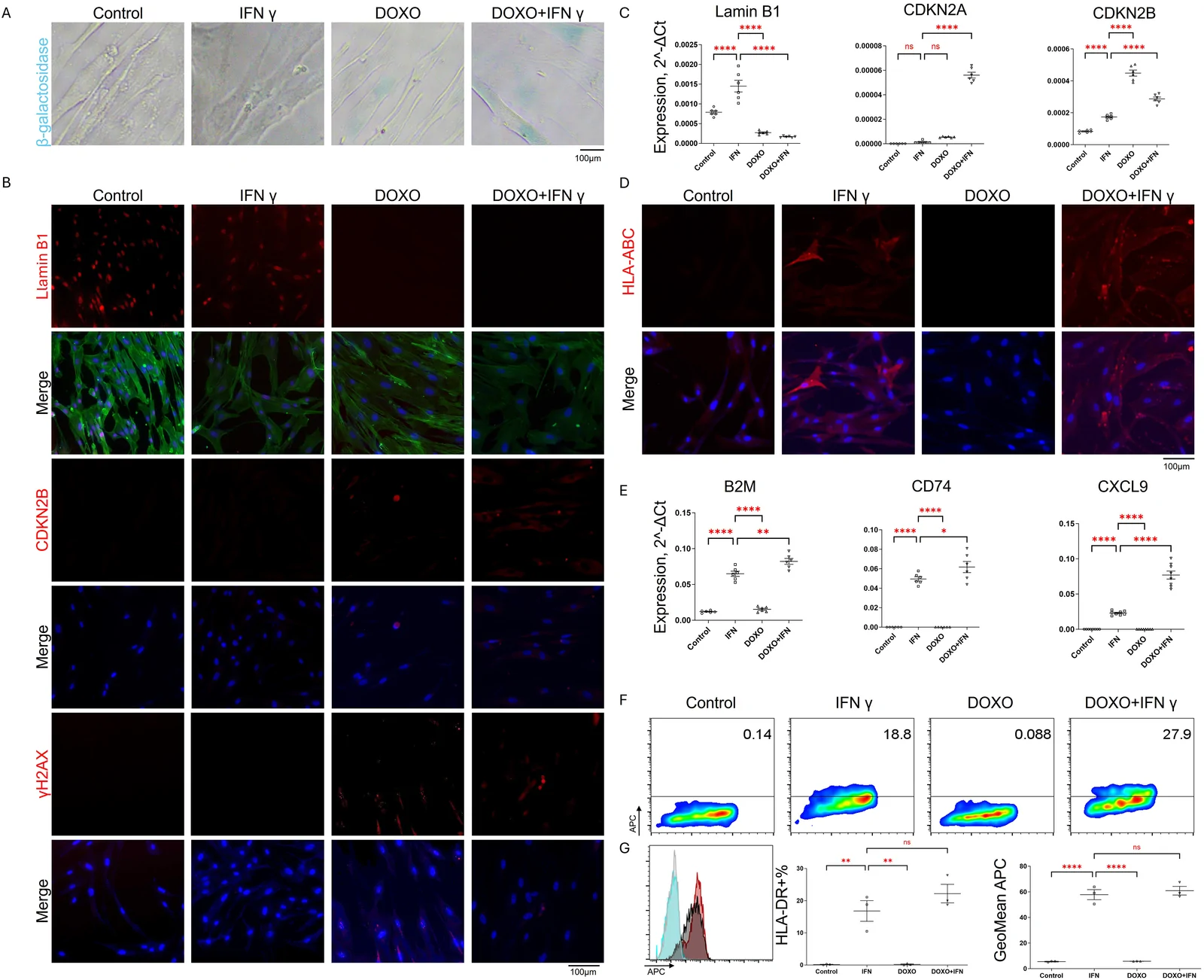
Senescence-like fibroblasts retain IFNγ-responsive antigen-presentation-associated and chemokine programs. **(A)** Representative SA-β-galactosidase staining of HFF-1 cells under control, IFNγ, DOXO, and DOXO+IFNγ conditions. **(B)** Representative immunofluorescence images showing Lamin B1, CDKN2B, γH2AX, and merged DAPI/phalloidin staining under the indicated conditions. **(C)** qPCR analysis of Lamin B1, CDKN2A, and CDKN2B expression. **(D)** Representative immunofluorescence images showing HLA-ABC expression under the indicated conditions. **(E)** qPCR analysis of B2M, CD74, and CXCL9 expression. **(F)** Representative flow-cytometry plots of surface HLA-DR staining after PI-based dead-cell exclusion. **(G)** Quantification of HLA-DR-positive fraction and HLA-DR APC geometric mean fluorescence intensity. For qPCR and flow-cytometry quantification, data were analyzed using two-way ANOVA with DOXO treatment and IFNγ stimulation as factors, followed by selected *post hoc* multiple comparisons. For IFNγ versus DOXO+IFNγ comparisons, adjusted P values were as follows: B2M, P = 0.0046; CD74, P = 0.0335; CXCL9, P < 0.0001; HLA-DR-positive fraction, P = 0.2552; HLA-DR APC geometric mean fluorescence intensity, P = 0.7098. For senescence-associated qPCR markers, IFNγ versus DOXO+IFNγ comparisons showed Lamin B1, P < 0.0001; CDKN2A, P < 0.0001; and CDKN2B, P < 0.0001. Asterisks indicate adjusted P values for selected *post hoc* comparisons shown in the figure: *P < 0.05, **P < 0.01, ***P < 0.001, ****P < 0.0001; ns, not significant.

DOXO-containing conditions showed multiple senescence- and DNA damage-associated features. SA-β-gal staining was low in control and IFNγ-treated cells but was more detectable after DOXO and DOXO+IFNγ treatment (**Figure 7A**). Immunofluorescence showed reduced Lamin B1 signal in DOXO-containing conditions, together with increased CDKN2B staining and increased γH2AX signal (**Figure 7B**). Quantification of fluorescence intensity supported these representative imaging patterns, with reduced Lamin B1 intensity and increased CDKN2B and γH2AX intensity in DOXO-containing conditions (**Supplementary Figure 6A**). DOXO-treated cells also appeared less confluent and more spread or flattened in representative fields, with DAPI staining suggesting more variable nuclear size and shape, although nuclear morphology was not quantified. qPCR supported this senescence-associated pattern: LMNB1 expression decreased from 7.89 × 10^-4 in control cells to 2.72 × 10^-4 after DOXO and 1.69 × 10^-4 after DOXO+IFNγ, while CDKN2A and CDKN2B were induced in DOXO-containing conditions (**Figure 7C**). CDKN2A showed its highest mean level in the DOXO+IFNγ group, whereas CDKN2B was highest after DOXO alone. Together, these results support that DOXO generated a senescence-like, DNA damage-associated fibroblast state under this experimental condition.

In contrast, antigen-presentation-associated and chemokine-related markers were mainly induced by IFNγ. HLA-ABC immunofluorescence was low in control and DOXO-alone cells but increased after IFNγ and DOXO+IFNγ treatment (**Figure 7D**). qPCR showed the same pattern for immune-related genes (**Figure 7E**). B2M increased from 0.0121 in control cells to 0.0649 after IFNγ and 0.0822 after DOXO+IFNγ, while DOXO alone produced only a small change. CD74 was minimally expressed in control and DOXO-alone cells but increased strongly after IFNγ and DOXO+IFNγ. CXCL9 showed a clear IFNγ-dependent pattern, remaining low in control and DOXO-alone cells but increasing to 0.0230 after IFNγ and 0.0770 after DOXO+IFNγ. Because the experimental design contained two factors, DOXO treatment and IFNγ stimulation, these quantitative readouts were reanalyzed using two-way ANOVA followed by selected *post hoc* comparisons. Direct comparison between IFNγ and DOXO+IFNγ showed that B2M, CD74, and CXCL9 were not reduced in the DOXO+IFNγ condition; instead, their expression was higher in DOXO+IFNγ than in IFNγ alone. Thus, IFNγ-induced antigen-presentation-associated and chemokine responses were preserved in DOXO-treated fibroblasts.

Flow cytometry confirmed this pattern at the surface protein level. The gating strategy included initial cell selection by FSC-A/SSC-A, singlet selection using SSC-W/SSC-H and FSC-W/FSC-H, PI-based exclusion of dead cells, and APC-channel thresholding using an APC negative control (**Supplementary Figure 6B**). HLA-DR-positive cells were rare in control and DOXO-alone conditions, with mean positive fractions of 0.147% and 0.199%, respectively (**Figures 7F, G**). IFNγ increased the HLA-DR-positive fraction to 16.8%, and DOXO+IFNγ increased it to 22.2%. HLA-DR geometric mean fluorescence intensity in the APC channel followed the same pattern, with low signal in control and DOXO-alone cells and higher signal in IFNγ-containing conditions. Direct *post hoc* comparison showed no significant reduction in either HLA-DR-positive fraction or HLA-DR APC geometric mean fluorescence intensity in DOXO+IFNγ cells compared with IFNγ alone. These results indicate that surface HLA-DR induction was primarily IFNγ-dependent and remained detectable after DOXO-induced senescence-like stress.

Overall, the fibroblast experiment addressed one specific point raised by the spatial analysis. DOXO induced a senescence-like fibroblast state, while IFNγ induced antigen-presentation-associated and chemokine-related programs. In the combined DOXO+IFNγ condition, these IFNγ-responsive immune programs remained detectable despite the DOXO-associated senescence-like features. This simplified *in vitro* system does not model the full multicellular psoriatic tissue environment and does not establish that fibroblast senescence generates the immune-aging spatial state. Instead, it provides orthogonal experimental support for the biological compatibility between senescence-like stromal stress and IFNγ-responsive immune activation, consistent with the spatial co-occurrence of stromal-aging and immune-inflammatory programs observed in the transcriptomic analyses.

## 4 Discussion

Aging skin is accompanied by inflammatory, immune, epithelial, and stromal remodeling, while psoriasis is a chronic immune-mediated inflammatory skin disease characterized by coordinated keratinocyte and immune-cell activation (11, 12). This study identifies spatially organized aging-associated programs within psoriatic skin and shows that these programs are not distributed as a single uniform aging signal. By integrating aging skin single-cell transcriptomics with psoriasis spatial transcriptomics, we found that aging-associated programs separated into distinct immune- and stromal-associated tissue contexts. Immune-aging activity was increased in lesional psoriasis and was associated with IFN response, antigen-presentation programs, chemokine activity, and immune-cell-enriched spatial contexts. In contrast, stromal-aging-high regions were characterized by extracellular matrix organization and fibro-inflammatory remodeling. External spatial transcriptomic analysis supported several features of this immune-aging-associated state, particularly its relationship with antigen-presentation and IFN-linked inflammatory activity. Finally, the HFF experiment showed that senescence-like stromal cells can retain IFNγ-responsive antigen-presentation-associated and chemokine programs, providing orthogonal experimental support for the biological compatibility between stromal stress and IFNγ-responsive immune activation, rather than direct validation of the full spatial tissue state. Together, these findings support a model in which aging-associated transcriptional programs are organized within inflammatory skin architecture through spatially distinct immune and stromal tissue states, rather than through a generalized increase in tissue aging.

The first step of the study was to define aging programs in skin before projecting them into psoriatic tissue. This analysis showed that aging was not captured by one homogeneous transcriptional program. Instead, old skin contained compositional changes across major skin compartments as well as transcriptional changes related to epithelial stress, stromal remodeling, and immune activation (7, 13). We therefore separated the aging signal into global old-up, stromal-aging, and immune-aging components. This compartment-informed strategy is consistent with prior single-cell studies showing that skin aging involves coordinated but cell-type-dependent changes across keratinocytes, fibroblasts, vascular cells, and immune populations. It is also biologically important because these compartments have different roles in inflammatory skin disease: keratinocytes shape barrier and cytokine responses, fibroblasts regulate extracellular matrix and tissue architecture, and immune cells provide inflammatory effector programs (14). A single aging score would merge these processes and make it difficult to distinguish whether an aging-associated signal in psoriasis reflects epidermal stress, stromal remodeling, immune activation, or changes in cell composition (15). Signature overlap analyses, overlap-removed scoring, and expression-matched random gene-set controls further contextualized the immune-aging program before spatial projection.

After defining compartment-informed aging signatures, the spatial analyses showed that these programs did not project onto psoriasis tissue as a uniform lesion-wide signal. Instead, immune-aging and stromal-aging scores localized to different marker-defined domains. Immune-aging and global old-up programs were highest in APC/immune-enriched and immune-inflammatory regions, whereas stromal-aging was strongest in fibro-inflammatory and ECM-stromal regions. This organization is consistent with the idea that psoriatic inflammation is maintained by spatially coordinated interactions among keratinocytes, immune cells, and fibroblasts, rather than by isolated cell populations acting independently (8). Prior single-cell and spatial studies have shown that keratinocytes and fibroblasts can amplify inflammatory responses in psoriasis, supporting the relevance of tissue-domain-level analysis rather than cell-type annotation alone (8). In our data, this concept was extended to aging-associated programs: the aging signal was not simply higher in lesional epidermis but partitioned into immune-inflammatory and stromal-remodeling tissue contexts. Because Visium spots can contain heterogeneous cell mixtures, these spatial domains should be interpreted as spot-level transcriptional tissue states rather than purified cell populations.

The separation between immune-aging-high and stromal-aging-high regions further suggests that aging-related programs are organized across more than one psoriatic tissue context. High-scoring regions were defined from score-based immune-aging and stromal-aging activity and then compared with marker-defined domains *post hoc*. The immune-aging-high regions carried IFN-linked chemokine, inflammatory, and immune-activation features, together with selected stress- or proliferation-associated markers, while the stromal-aging-high regions were dominated by extracellular matrix organization, collagen biology, and fibro-inflammatory remodeling (16). This distinction is important because it avoids treating “aging” as a single modifier of psoriasis. An immune-aging signal may relate more closely to IFN-associated inflammatory activity, antigen-presentation programs, and immune-cell-enriched tissue contexts, whereas a stromal-aging signal may reflect matrix remodeling, tissue stiffness, wound-healing-like responses, or altered fibroblast organization (17). These are biologically different processes, and they may have different implications for lesion persistence, tissue remodeling, or therapeutic response. Thus, the spatial analysis connects aging biology with psoriatic tissue organization without reducing the result to either keratinocyte hyperproliferation alone or immune-cell infiltration alone.

In the discovery cohort, immune-aging activity was increased in lesional sections and was associated with IFN-linked inflammatory activity at the section level. Section-level summaries were used to relate immune-aging activity to tissue-scale inflammatory programs while retaining patient identity in the visualizations. The strongest section-level association was observed between immune-aging activity and IFN gamma response, and this relationship was preserved with the overlap-removed immune-aging score. These findings support a tissue-level relationship between immune-aging activity and interferon-responsive inflammatory architecture (18). Threshold-defined high-region fraction provided a complementary supporting readout and showed weaker lesional separation than the continuous immune-aging score. Taken together, these results suggest that immune-aging activity is part of a broader lesional tissue state in which immune-associated programs and inflammatory activity co-occur (19). The spatial data remain observational, and these associations should be interpreted as tissue-level co-organization rather than a causal relationship with lesion formation.

The external spatial cohort provided an important assessment of how this immune-aging-associated pattern behaved across datasets with different sample composition. Section-level immune-aging activity showed a modest increase in cutaneous psoriasis lesional sections, and the overlap-removed immune-aging score showed a similar pattern. Across the full external dataset, immune-aging activity was positively associated with IFN gamma response and most strongly associated with antigen-presentation activity (20, 21). Healthy sections also contained measurable immune-aging activity, the psoriatic arthritis subset showed a less consistent lesional pattern, and cytokine/chemokine activity differed from the discovery cohort. These findings refine the interpretation of the immune-aging-associated state: it should not be viewed as a generic cytokine-high or psoriasis-specific score. Instead, the most consistent component was an antigen-presentation- and interferon-associated tissue program that can appear across inflammatory or remodeling skin contexts, with its abundance and module relationships varying by disease subtype and cohort structure (22).

The cellular-context analysis further supports the interpretation that the immune-aging-high spatial state is a multicellular tissue context rather than a proxy for a single cell type (8, 23). Projection of aging and inflammatory programs onto the GSE130973 aging skin single-cell reference showed that immune-aging activity was strongest in immune cells, while the global old-up program was more prominent in keratinocytes (24). At the same time, IFN response, antigen-presentation, and cytokine/chemokine programs were distributed across immune and non-immune compartments, including stromal, endothelial, and pericyte/smooth muscle populations (25). This pattern fits the spatial data, where score-defined immune-aging-high spots were enriched for immune-associated marker programs, including T-cell, cytotoxic-lymphocyte, macrophage/monocyte, and dendritic/APC programs. These spots also showed co-activation of IFN gamma response, CXCL9/CXCL10 signaling, antigen presentation, macrophage/DC activation, T-cell cytotoxicity, and TNF/IL1/NF-κB-related programs. Together, these results support a local immune-cell-enriched inflammatory context in which multiple immune-associated programs co-occur within the same tissue region. Because Visium spots contain mixed cellular signals, the biologically useful interpretation is not a purified cell identity, but a spatially organized tissue context.

The fibroblast experiment provided a reductionist test of one component of this model. The spatial data suggested that stromal-aging and immune-inflammatory programs can occur in related tissue contexts, but spatial transcriptomics alone cannot determine whether stromal cells with senescence-associated features remain responsive to inflammatory cytokines. In HFFs, DOXO induced a senescence-like DNA damage-associated state, whereas IFNγ induced antigen-presentation-associated and chemokine-related programs (26, 27). In the combined DOXO+IFNγ condition, IFNγ-responsive markers, including HLA-ABC, B2M, CD74, CXCL9, and surface HLA-DR, remained detectable despite the presence of DOXO-associated senescence-like features (28, 29). This experiment does not reproduce the full psoriatic tissue environment. Its contribution is narrower: it shows that a senescence-like stromal state remains compatible with IFNγ-responsive antigen-presentation-associated and chemokine programs. Thus, the *in vitro* data support the compatibility between senescence-like stromal stress and IFNγ-responsive immune programs, while leaving open whether similar co-occurrence *in vivo* involves fibroblasts, vascular cells, myeloid cells, keratinocytes, or combinations of these populations.

### 4.1 Limitations and future directions

Several limitations should be considered. First, the discovery spatial cohort contained a limited number of patients and tissue sections. We therefore used section-level summaries for the main comparisons and correlations to reduce spot-level pseudoreplication, but larger cohorts will be needed to test patient-to-patient variability and to determine whether the immune-aging-associated spatial state is associated with clinical features such as disease duration, chronological age, disease severity, or treatment status. Chronological age was used to define aging-associated signatures in the GSE130973 aging skin scRNA-seq reference; however, the psoriasis spatial cohorts were not powered for reliable age-stratified subgroup analysis. Patient-level age metadata were not uniformly available in a format that supported age-stratified spatial comparisons across the psoriasis cohorts. Future studies using larger psoriasis cohorts with complete age and clinical metadata will be needed to determine whether the immune-aging- and stromal-aging-associated spatial programs identified here vary according to patient age within psoriasis. Because only three patients were available in the discovery spatial cohort, section-level statistical analyses should be interpreted as exploratory, even when nominal P values were reported.

Second, the spatial analysis was based on spot-level transcriptomic data. Because each Visium spot can contain multiple cells, the spatial domains and high-scoring region classes should be interpreted as tissue states rather than purified cellular identities (30). The aging skin single-cell reference projection, marker-based enrichment, overlap-removed scoring, and immune/APC marker-adjusted residual analyses helped provide cellular context and sensitivity testing, but they do not replace formal high-resolution spatial cell typing, direct cell-cell interaction mapping, or experimental lineage-specific validation. We evaluated formal reference-based deconvolution as a potential cell-composition adjustment, but the processed spatial object used for the full discovery cohort did not contain raw integer UMI counts suitable for robust RCTD-based deconvolution. The available integer count matrix covered only a subset of the cohort, and previously available broad-class deconvolution outputs did not resolve the APC/myeloid, T/NK, or B/plasma compartments needed to address immune-subtype-specific confounding. Therefore, we did not use formal deconvolution as a primary cohort-level analysis. Instead, we used overlap-removed scoring, expression-matched random gene-set controls, marker-based cellular-context scoring, and immune/APC marker-adjusted residual analysis as sensitivity analyses. These analyses provide cellular-context support but cannot assign the immune-aging signal to a specific cell type of origin.

Third, the aging-associated signatures were defined from an independent aging skin single-cell dataset and then projected into psoriasis spatial data. This strategy allowed us to use normal skin aging as a reference framework, but it also means that differences in dataset composition, sequencing depth, tissue processing, and annotation could influence the projected scores. Expression-matched random gene-set controls and overlap-removed immune-aging analyses supported the specificity of the projected signatures, but these analyses cannot fully exclude all dataset- or composition-dependent effects.

Fourth, the external spatial cohort provided supportive but incomplete concordance. The elevation of immune-aging activity in cutaneous psoriasis lesional sections and the association with antigen-presentation and IFN-linked activity were consistent with the discovery analysis, but the cytokine/chemokine relationship differed across cohorts and immune-aging-high spots were also detectable in healthy and psoriatic arthritis sections. These findings support a reproducible spatial inflammatory component, but not one that is exclusive to psoriasis lesions or equivalent to a generic inflammatory score.

Finally, the HFF-1 experiment was intentionally reductionist. DOXO-treated fibroblasts provided a senescence-like DNA damage-associated stromal model, and IFNγ stimulation tested inflammatory responsiveness in that context (31). However, this system does not reproduce psoriatic tissue complexity, including keratinocyte–fibroblast–immune-cell interactions, vascular components, chronic cytokine exposure, or spatial organization *in vivo*. Fresh patient biopsies, primary psoriasis-derived fibroblasts, and matched histological protein validation were not included in this study. Because the transcriptomic analyses were based on public datasets, matched tissue specimens from the same spatial cohorts were not available for additional protein-level validation or primary-cell isolation. Such experiments would require a separate prospective clinical validation study with dedicated ethics approval, patient recruitment, paired lesional and non-lesional biopsy collection, tissue processing, primary-cell culture, histological or spatial protein assays, and dedicated resources. Therefore, the HFF-1 experiment should be interpreted as a controlled reductionist assay supporting the compatibility between senescence-like stromal stress and IFNγ-responsive antigen-presentation-associated and chemokine programs, rather than as direct validation of the spatial transcriptomic model in patient tissue.

In conclusion, this study shows that aging-associated transcriptional programs are spatially organized within psoriatic skin rather than appearing as a uniform aging signal. Immune-aging activity was increased in lesional tissue and was associated with IFN response, antigen-presentation, chemokine signaling, and immune-cell-enriched spatial contexts, whereas stromal-aging-high regions reflected ECM organization and fibro-inflammatory remodeling. These findings support a model in which aging-related immune and stromal programs mark distinct but related tissue contexts within inflammatory skin. Future studies using larger patient cohorts, higher-resolution spatial technologies, patient-derived experimental systems, and perturbation-based approaches will be needed to determine whether and how these aging-associated spatial states relate to lesion persistence, inflammatory memory, or treatment response. More broadly, these findings are consistent with current frameworks linking aging hallmarks to age-related disease progression and emphasizing keratinocyte–immune-cell crosstalk in psoriasis (32, 33).

## Data Availability

The public datasets analyzed in this study are available in the NCBI Gene Expression Omnibus under accession numbers GSE130973, GSE206391, and GSE202011. The in vitro HFF-1 data generated in this study are available from the corresponding authors upon reasonable request.

## Supplementary material

Supplementary Figure 1 Aging signature definition, overlap analysis, and random gene-set controls. **(A)** UMAP visualization and broad compartment annotation of the aging skin single-cell dataset. **(B)** Marker support for broad cell-type annotation and donor-level cell-type composition across young and old samples. **(C)** Relationship between the strict 683-gene global old-up score and the broader 1,012-gene old-biased score at the donor-compartment level. **(D)** Detected gene counts for global old-up, immune-aging, and stromal-aging signatures across the aging scRNA-seq dataset and both spatial transcriptomic datasets. **(E)** Overlap between the immune-aging signature and canonical immune/inflammatory modules. **(F)** Expression-matched random gene-set controls for the aging-associated signatures.

Supplementary Figure 2 Spatial projection and sensitivity analyses for aging-associated signatures in the discovery psoriasis spatial cohort. **(A)** Expanded spatial states contributing to the five marker-defined spatial domains used in the main analysis.**(B)** Marker-defined spatial domain maps across all discovery sections.**(C)** Spatial projections of global old-up, immune-aging, and stromal-aging scores across all discovery sections. **(D)** Correlation between the original immune-aging score and the overlap-removed immune-aging score after removing the three canonical immune-overlap genes CXCL2, CXCL3, and LYZ. **(E)** Representative spatial projection of the overlap-removed immune-aging score. **(F)** Marker-based immune and cellular context across marker-defined spatial domains. Cellular-context scores were calculated using predefined marker programs for keratinocyte, fibroblast/stromal, APC/myeloid, T cell, NK/cytotoxic, B/plasma cell, endothelial, and pericyte/VSMC compartments. **(G)** Immune/APC marker-adjusted immune-aging residual score. Residual scores were obtained by regressing the immune-aging score against immune/APC-related marker programs and summarizing the residual signal at the domain level. **(H)** Representative spatial projection of the immune/APC marker-adjusted residual score.

Supplementary Figure 3 Sensitivity analyses for score-defined aging-high spatial regions. **(A)** Threshold sensitivity analysis for score-defined high-region assignments. High regions were defined using top 10%, 15%, or 20% score cutoffs, and marker-defined domains were overlaid post hoc to summarize domain distribution among high-scoring spots. **(B)** Overlap-removed immune-aging high-region sensitivity. Immune-aging-high regions were redefined using the 112-gene overlap-removed immune-aging score, and high-region assignments were compared with the original immune-aging score-based definition. **(C)** Within-domain high-versus-low program gradient. Immune-aging and stromal-aging high-versus-low comparisons were performed within immune-associated or stromal-associated domains to reduce circularity from domain-level definitions. **(D)** All-section maps of score-defined aging-high regions across the discovery spatial cohort.

Supplementary Figure 4 Supporting analyses for score-defined immune-aging-high regions and section-level inflammatory activity. **(A)** Score-defined high-region fraction by lesion status. **(B)** Correlations between score-defined immune-aging-high region fraction and inflammatory module activity. **(C)** Exploratory tissue-level co-occurrence between mean immune-aging score and psoriatic keratinocyte-associated programs. **(D)** Patient-level paired deltas for the main **Figure 4** readouts. **(E)** All-section maps of **Figure 4** score-defined high regions.

Supplementary Figure 5 Supporting analyses of threshold-defined immune-aging-high spot fraction and external cohort context. **(A)** Immune-aging-high spot fraction across external cohort groups. High spots were defined using the threshold-based immune-aging-high classification applied to the external cohort. Each point represents one tissue section, and point size indicates retained spot count. **(B)** Section-level correlations between immune-aging-high spot fraction and inflammatory module activity, including IFN gamma response, cytokine/chemokine activity, and antigen-presentation activity. These threshold-based analyses were used as supporting analyses for the continuous section-level immune-aging activity results. **(C)** Healthy and psoriasis-only analysis of section-level immune-aging activity. This analysis shows immune-aging activity across healthy, psoriasis non-lesional, and psoriasis lesional sections after excluding psoriatic arthritis sections. **(D)** Separate psoriatic arthritis context analysis. Immune-aging-high spot fraction and immune-aging score are shown for healthy, psoriatic arthritis non-lesional, and psoriatic arthritis lesional sections as supporting context analyses. **(E)** Representative maps of threshold-defined immune-aging-high spots in selected external sections. Blue spots indicate immune-aging-high spots, and gray spots indicate non-high spots.

Supplementary Figure 6 Quantification of immunofluorescence signals and flow-cytometry gating strategy for the HFF-1 experiment. **(A)** Quantification of fluorescence intensity for Lamin B1, CDKN2B, γH2AX, and HLA-ABC staining under control, IFNγ, DOXO, and DOXO+IFNγ conditions. Data are shown as individual measurements with mean ± SEM. Statistical testing was performed using two-way ANOVA with DOXO treatment and IFNγ stimulation as factors, followed by selected post hoc comparisons. **(B)** Representative flow-cytometry gating strategy for HLA-DR analysis. Cells were first selected by FSC-A/SSC-A, followed by SSC and FSC singlet gates. PI-positive dead cells were excluded, and HLA-DR positivity in the APC channel was defined using an APC negative control. Representative HLA-DR gates are shown for control, IFNγ, DOXO, and DOXO+IFNγ conditions.

Supplementary Table 1 global_old_up_signature_genes.

Supplementary Table 2 stromal_aging_signature_genes.

Supplementary Table 3 immune_aging_signature_overlap_annotation.
